# A Multilocus Approach to Understanding Historical and Contemporary Demography of the Keystone Floodplain Species *Colossoma macropomum* (Teleostei: Characiformes)

**DOI:** 10.3389/fgene.2018.00263

**Published:** 2018-08-14

**Authors:** Maria da Conceição Freitas Santos, Tomas Hrbek, Izeni P. Farias

**Affiliations:** ^1^Departamento de Biologia, Universidade do Estado do Amazonas, Manaus, Brazil; ^2^Laboratório de Evolução e Genética Animal, Departamento de Genética, Universidade Federal do Amazonas, Manaus, Brazil

**Keywords:** tambaqui, microsatellites, mitochondrial DNA, genetic variability, gene flow, genetic structure, Amazon basin

## Abstract

We studied the natural populations of a flagship fish species of the Amazon, *Colossoma macropomum* which in recent years has been suffering from severe exploitation. Our aim was to investigate the existence or not of genetic differentiation across the wide area of its distribution and to investigate changes in its effective population size throughout its evolutionary history. We sampled individuals from 21 locations distributed throughout the Amazon basin. We analyzed 539 individuals for mitochondrial genes (control region and ATPase gene 6/8), generating 1,561 base pairs, and genotyped 604 individuals for 13 microsatellite loci obtaining, on average, 21.4 alleles per locus. Mean *H*_*E*_ was 0.78 suggesting moderate levels of genetic variability. AMOVA and other tests used to detect the population structure based on both markers indicate that *C. macropomum* comprises a single and large panmitic population in the main channel of the Solimões-Amazonas River basin, on the other hand localities in the headwaters of the tributaries Juruá, Purus, Madeira, Tapajós, and localities of black water, showed genetic structure. The greatest genetic differentiation was observed between the Brazilian Amazon basin and the Bolivian sub-basin with restricted genetic flow between the two basins. Demographic analyzes of mitochondrial genes indicated population expansion in the Brazilian and Bolivian Amazon basins during the Pleistocene, and microsatellite data indicated a population reduction during the Holocene. This shows that the historical demography of *C. macropomum* is highly dynamic. Conservation and management strategies should be designed to respect the existing population structure and minimize the effects of overfishing by limiting fisheries *C. macropomum* populations.

## Introduction

The Amazon basin holds the largest diversity of fishes in the world. It is estimated that approximately 2,411 fish species occur there (Reis et al., 2016), with 1,089 species being endemic. Aquatic biodiversity of the Amazon basin is thought to be the consequence of diversification of modern fauna that occurred mainly during the Miocene (Lovejoy et al., 2010), driven, to a large extent by the establishment of the current hydroscape. Amazonian rivers also drain three principal geological formations, the Andes and the Guyana and Brazilian Shields, with consequences for the physicochemical properties of the waters draining these geological formations. Thus some of these rivers present physical barriers which limit geneflow between different sections of the river, further acting as agents of divergence (Hoorn et al., 2010). Naturally, all of these forces interact, producing an amazingly diverse ichthyofauna. Part of this ichthyofauna is also exploited as a fisheries resource that represent the production base of an economic sector that contributes more than US$ 200 million per year to the economy of the Brazilian Amazon basin (Barthem and Fabré, 2003). *Colossoma macropomum* (tambaqui) is on the top of the list of most important commercial species. This species also has an important ecological role, as it is an important disperser of seeds of trees and shrubs of the Amazonian floodplain (Araújo-Lima and Goulding, 1998). For all its commercial importance, this species has suffered over-exploitation of its natural stocks over the last years and today, juveniles account for most of the catch (Barthem and Goulding, 2007). The average size of the fish landed and sold in the main markets in the Amazon suggests that many individuals are captured before reaching sexual maturation, which occurs in females between 50 and 55 cm in length, at an estimated mean age of 3 years (Goulding and Carvalho, 1982; Isaac et al., 1996) based on the length/age relationship estimated from Bertalanffy's model by Isaac and Ruffino (1996).

*Colossoma macropomum* is found throughout almost the entire length of the Amazon River and most of its affluents, as well as in the Orinoco basin (Araújo-Lima and Goulding, 1998). Thus, the species is found in the three main Amazonian water types (white, clear, and black), as well as upstream and downstream of geographic barriers such as rapids (Goulding et al., 2003). *Colossoma macropomum* is thus an idea candidate for the study of and the understanding of the structuring patterns in the Amazon basin, which are important for the implementation of science-driven conservation measures.

Previous studies have found a high degree of genetic variability of populations of *C. macropomum* (Santos et al., 2007; Farias et al., 2010), indicating that overfishing has not yet affected genetic diversity of wild populations, nor were signals of population reduction detectable. The authors suggested that the absence of a genetic sign of population reduction was likely related to the large effective population size of the species. Moreover, *C. macropomum* has migratory behavior and moves through the rivers of the Amazon seasonally for the purposes of feeding and breeding (Araújo-Lima and Ruffino, 2004). The behavior of *C. macropomum* and the hydrological dynamic of the floodplain habitat it predominantly occupies may partially explain the panmixia reported for this species. This apparent lack of population structuring is found throughout the mainstream of the Amazon basin, with the exception of individuals found upstream of the series of rapids delimiting the Bolivian sub-basin from the Amazon basin (Farias et al., 2010). The authors also suggested that populations of *C. macropomum* from Bolivian sub-basin were largely demographically stable, while the Brazilian Amazon basin populations evidenced a historical population growth from the Pleistocene onward.

Knowledge of changes of effective population sizes of *C. macropomum* is important for understanding the demography of the species. In addition, robust estimates of population differentiation, are important for implementing conservation and management strategies. Therefore, the aim of the present study was to test the two hypotheses raised in previous studies of *C. macropomum* using samples from the entire area of distribution of *C. macropomum* in the Amazon basin, and using both nuclear-encoded microsatellites and mtDNA genes sequences. As the first hypothesis we test if *C. macropomum* populations are differentiated, considering: (i) samples of the mainstream of the Amazon River, as well as eight of its main tributaries; (ii) samples of all three major water types of the Amazon (white, clear, and black water) based on the classifications of Sioli (1984) and Venticinque et al. (2016); (iii) samples of water upstream and downstream of rapids in the Madeira and Tapajós rivers. In the second hypothesis, historical and contemporary demographic approaches were used to test if *C. macropomum* underwent changes in the effective population size throughout its evolutionary history in the Amazon basin.

## Materials and methods

### Samples and data collection

**Figure 1:**
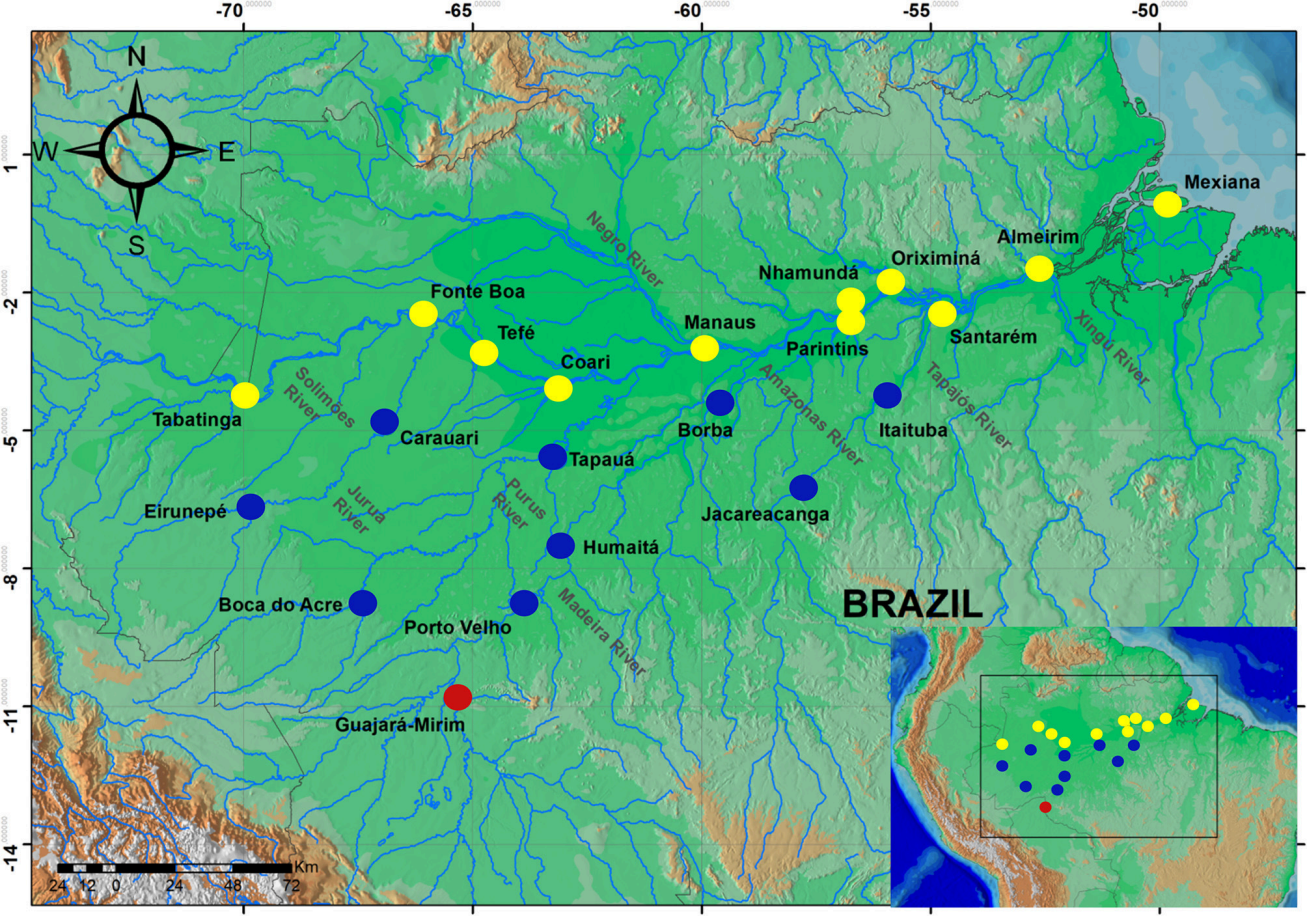
Map of the Amazon basin showing sampled localities. Circles represent localities in the mainstream of Amazon River (yellow), tributaries of the Amazon River (blue), and the locality of Bolivian sub-basin (red).

A total of 637 samples of *Colossoma macropomum* were collected directly from artisanal fishers at 21 localities within the Brazilian Amazon basin and one locality in the Bolivian sub-basin (Figure 1). The adipose fin or fragment of muscle tissue was removed from between 20 (localities within the Brazilian Amazon Basin) and 69 individuals (within the Bolivian sub-basin) and then preserved in 100% ethanol for subsequent laboratory analyzes.

Total genomic DNA was extracted using Proteinase K/Phenol-chloroform/isoamyl alcohol protocol and precipitated with 70% ethanol (Sambrook et al., 1989). Approximately 50 to 100 ng of genomic DNA was used as a template for PCR reactions. We amplified the mitochondrial DNA control region (mtDNA control region) and the ATPase subunits 6 and 8, using the primers Chara_LDloop and Chara_RDloop; CMF2 and CMR2 (control region) and ATP 8.2_L8331 and CO3.2_H9236 (ATPase genes) listed in Supplementary Table S1. The PCR reactions for the two regions were performed in a final volume of 15 μL containing 1.5 μl of the forward and reverse primer (2 mM), 1.5 μl of buffer (Tris-KCL 200 mM, pH 8.5), 1.5 μL of 25 mM MgCl, 1.5 μL of 25 mM dNTP, 0.3 μL of 5 U/μL Taq polymerase and 6.2 μL ddH_2_O. PCR conditions (for control region and ATPase gene) were as follows: denaturation at 94°C for 60 s, primer annealing at 50°C for 30 s, primer extension at 68°C for 90 s, followed by a final extension at 68°C for 5 min. The first three steps were repeated 35 times.

Purification of the PCR products was performed using ExoSAP (Exonuclease Enzymes and Shrimp Phosphatase Alkaline). The samples were sequenced using the BigDye terminator v3 kit (ThermoFisher), following the manufacturer's protocol. Due to the size of the control region of *C. macropomum* (approximately 1,100 bp), each sample was sequenced in two steps, using the CMF2 (forward) and CMR2 (reverse) internal primers (Supplementary Table S1). For the ATPase gene only the primer ATP 8.2 (forward) was used. The precipitated product was resolved in the ABI 3130xl DNA Analysis System sequencer (ThermoFisher), according to the manufacturer's standard protocol.

Microsatellite genotypes were generated using a multiplex design (Supplementary Table S2) using 13 pairs of primers developed by Santos et al. (2009) for *C. macropomum*. The amplification conditions for each multiplex were: For three pairs of primers: 1.5 μl MgCl_2_ (25 mM), 1.5 μl dNTPs (10 mM), 1.5 μl 10x buffer (100 mM Tris-HCl, 500 mM KCl), 1.0 μL of each forward primer containing one of the two M13 tails (2 μM), 1.5 μL of each reverse primer, 1.5 μL of fluorescence-labeled M13f (FAM) primer, 0.7 μL of primer fluorescence-labeled M13r (HEX), 0.8 μl Taq DNA Polymerase (5 U/μl) and 1 μl DNA (50–100 ng), with a final volume of 14.5 μl. For two pairs of primers: 3.0 μl ultra pure water, 1.5 μl MgCl_2_ (25 mM), 1.5 μl dNTPs (10 mM), 1.5 μl 10x buffer (100 mM Tris-HCl, 500 mM KCl), 1.0 μL of each forward primer containing one of the M13 tails (2 μM), 1.5 μL of each reverse primer, 1.5 μL of the fluorescently labeled M13f primer (FAM), or 0.7 μL of fluorescently labeled M13r primer (HEX), 0.6 μl of Taq DNA Polymerase (5 U/μl), and 1 μl of the DNA (50–100 ng), with a final volume of 14 μl.

PCR conditions were as follows: denaturation at 94°C for 20 s, primer annealing at 60–65°C (depending on the primer combination) for 20 s, and extension at 68°C for 30 s, repeated for 30 times, followed by another cycle for annealing the M13 primers with the following conditions: denaturation at 94°C for 20 s, annealing of the M13 fluorescence-labeled primer at 50°C for 20 s, and extension at 68°C for 30 s, repeated for 20 times, with final extension of 30 min at 68°C.

For the genotyping reaction the PCR products were diluted to between 10–50 μL with ultra-pure water depending on the intensity of PCR products on an agarose gel. For each 1 μl of diluted product, 8.0 μL of Hi-Di formamide (ThermoFisher, Inc.), and 1.0 μL 6-carboxy-X-rhodamine (ROX) size standard from DeWoody et al. (2004) were added. The samples were genotyped in ABI 3130xl automatic sequencer (ThermoFisher, Inc.) and allele sizes (in base pairs) were estimated in *GeneMapper*™ software version 4.0 (ThermoFisher, Inc.). Matrix of genotypes is available at https://github.com/legalLab/datasets.

The sequences of the control region and subunits 6 and 8 of the ATPase gene were verified, edited and aligned in the program BIOEDIT v7.0.5 (Hall, 1999). The ATPase genes were translated into hypothetical amino acids in the program MEGA 6.0 (Tamura et al., 2013) to verify the presence of any unexpected stop codons. Sequences were deposited in the GenBank under accession numbers MH514288–MH514827 for control region and MH520124–MH520663 for ATPase genes.

### Mitochondrial DNA analyses

The existence of population structure was tested for using the Analysis Molecular Variance (AMOVA) implemented in the program ARLEQUIN v3.5 (Excoffier and Lischer, 2010). We analyzed three datasets: (1) all 21 locations were analyzed as a single hierarchical level; (2) the Guajará-Mirim (Bolivian basin) locality was removed from the data matrix; 3) tributaries vs. locations of the main channel, of Amazon River. Pairwise Φ_*ST*_ were also estimated, and statistical significance was corrected for multiple comparisons (Rice, 1989). We also tested for population structuring using the Spatial Analysis of Molecular Variance (SAMOVA) (Dupanloup et al., 2002), and the Bayesian Analysis of Genetic Population Structure (BAPS) (Mantel, 1967; Corander and Tang, 2007). Spatial structuring was tested using the Mantel test as implemented in ARLEQUIN v3.51 (Excoffier and Lischer, 2010).

In order to investigate patterns of change in *C. macropomum* historical effective population sizes, we carried out a Bayesian Skyline plot analyses in the program BEAST v.1.8.4 (Drummond and Rambaut, 2007). We collected 50,000,000 Monte Carlo Markov Chain steps (MCMC), discarde the first 5,000,000 steps as burnin, and subsequently sampled every 1,000th step, retaining 45,000 topologies. The HKY85 (Hasegawa et al., 1985) model of molecular evolution was selected as the best fitting model in the program Modeltest. We estimated a genetic network of mtDNA haplotypes from all samples using Network (http://www.fluxus-engineering.com/) using the median-joining algorithm.

To convert the results of the coalescent analyzes into years and effective number of individuals, we assumed a three-year generation time (Goulding and Carvalho, 1982; Isaac and Ruffino, 1996), and a rate of molecular evolution of 2.0 × 10^−8^ mutations per site and per year (Farias et al., 2010).

The Tajima's *D* (Tajima, 1989) and the Fu's *Fs* (Fu, 1997) tests were used to examine whether populations are at a mutation-drift equilibrium assuming no selective differences among haplotypes. Both tests were performed using the program ARLEQUIN v3.5 (Excoffier and Lischer, 2010). Demographic history may also be inferred from frequency distribution of pairwise haplotype differences. In populations that are at a demographic equilibrium, the distribution of differences are generally multimodal, while populations that have undergone recent expansion or reduction typically have a unimodal distribution (Slatkin and Hudson, 1991). In order to distinguish population reduction and expansion, we used the results of two tests. The first test evaluates the distribution of the sum of the squares of differences (SSD) between the mismatch distribution observed for each locality and the expected distribution for a null expansion model, where significant values for SSD indicate deviations from the population expansion model (Schneider and Excoffier, 1999). The other test is based on the Harpending inequality index (Hri = *r*) (Harpending, 1994), which quantifies the variance of the mismatch distribution, assuming that the mismatch distribution is unimodal. These analyzes were performed in the programs DNASP v5.0 (Librado and Rozas, 2009) and ARLEQUIN v3.5 (Excoffier and Lischer, 2010); significance was tested via 10,000 permutations with a *P* = 0.05 cut-off.

### Microsatellite DNA analyses

The data matrix with allele sizes was verified for the occurrence of null alleles, allelic stutter, and large allele dropout in the program MICRO-CHECKER (Van Oosterhout et al., 2004) The number of alleles (A), observed (*H*_*O*_) and expected (*H*_*E*_) heterozygosities, gene diversity (*h*), nucleotide diversity (π), linkage disequilibrium (LD) between pairs of loci and the Hardy-Weinberg equilibrium (HWE) were calculated. All these parameters were estimated using ARLEQUIN v3.5 (Excoffier and Lischer, 2010). Considering that some of these estimates suffer influence of sample size (Leberg, 2002), we implemented a rarefaction analysis and calculated allelic richness (AR) and private allelic richness (PAR) in the program HP-Rare (Kalinowski, 2005), so that the number of alleles and allele richness estimates could be compared between localities. Additionally, we estimated the inbreeding coefficient (*F*_*IS*_) for each sample. The effective population size (*Ne*) for each population was estimated using the LD method (Waples and Do, 2008) as implemented in NeEstimator 2.0 (Do et al., 2014). The *Ne* estimates are equivalent to the effective number of breeders that produced offspring during a certain period of time and assuming that sample sizes are not representative of the entire generation (Palstra and Fraser, 2012). In all instances, significance levels for tests involving multiple comparisons were adjusted using the sequential Bonferroni correction (Rice, 1989).

The overall genetic structuring was estimated using the analysis of molecular variance (AMOVA—Excoffier et al., 1992) performed in the program Arlequin v3.5 (Excoffier and Lischer, 2010). We also analyzed three datasets: (1) all 21 locations were analyzed as a single hierarchical level; (2) the Guajará-Mirim (Bolivian basin) locality was removed from the data matrix; (3) tributaries vs. locations of the main channel of Amazon River. Genetic differentiation between pairs of populations was estimated using *F*_*ST*._ Additionally, pairwise genetic differentiation between populations was estimated using Hedrick's *G*_*ST*_, (Hedrick, 2005) based on the empirical Bayes (EB) *G*_*ST*_ estimator (Kitada et al., 2007) suitable for high gene flow species (Kitada et al., 2017), using the FinePop 1.3.0 package (Kitada et al., 2017) implemented in the R statistical language (R Development Core Team, 2011).

We used SAMOVA (Dupanloup et al., 2002) to infer spatial population structure and STRUCTURE v2.3.4 (Pritchard et al., 2000) to identify biological populations. For STRUCTURE analyses we used the admixture and correlated allelic frequencies models with and without location prior, and we tested one to 20 groups (*K* = 1–20). The analysis was run with 1,000,000 MCMC step, discarding the first 100,000 steps. The Isolation by Distance (IBD) was tested via a correlation between genetic and geographical distance using the Mantel test implemented in the Software Arlequin (Excoffier and Lischer, 2010). In addition, we also used a multivariate approaches implementing the Discriminant Analysis of Principal Components (DAPC; Jombart et al., 2010) to cluster genotypes using the R package Adegenet (Jombart, 2008).

Coalescent analyses implemented in the program IMa2 (Hey and Nielsen, 2007) were used to partition allele sharing between populations due to ongoing geneflow and ancestral haplotype sharing. We estimated the parameters *t* (splitting time), *m* (migration rates), and theta (θ) where θ = *2 Ne*μ. We sampled 20,000,000 Monte Carlo Chain Markov Chain Monte Carlo (MCMC) generations after discarding the first 1,000,000 generations as burn-in. Two independent runs were carried out with different starting points, in order to verify convergence. The two independent runs converged and thus were combined and the parameters θ, *m*, and *t* were estimated. Then, these were converted into demographic parameters: contemporary effective population size, number of migrants per generation, and time of divergence of the populations in generations. The analyses using IMa2 were performed with pairs of sampling sites located at the geographic extremes along the main channel of the Amazon River and between upstream and downstream localities of principal tributaries. We also estimated geneflow using the program MIGRATE 3.1.6 (Beerli and Felsenstein, 2001), where for diploid data θ = 4 *Ne*μ and *M* = m/μ migration rate ratio and mutation rate. For the Bayesian analysis, we ran ten short chains, sampling each chain 10,000 times. Then we ran six long chains of 2,000,000 steps, sampling each chain 200,000 times and discarding the first 2,000 samples. The runs were replicated, and the convergence between the chains was evaluated using the Gelman-Rubin statistic implemented in the program. We estimated the historical migration rates (*M*) between the localities and the relative number of migrants per generation *Nm* = Mθ/2. To convert the results into biological information, we assumed a 3-year generation time for *C. macropomum* (previously justified with mtDNA data), and a mutation rate μ = 5 × 10^−4^ (mean rate of evolution of microsatellites, Di Rienzo et al., 1994).

To detect, quantify, and date the historical and contemporaneous demographic changes in *C. macropomum* populations we implemented the coalescent sampler implemented in the program MSVar v1.3 (Beaumont, 1999; Storz et al., 2002). We ran 11 independent parallel chains sampling every 1,000th proposal to collect 20,000 proposals in the MCMC chain in each parallel run. Priors for current and historical population size means and variances were equal, and variances encompassed three orders of magnitude. Prior for mean time of population size change was set at 1,000 with variance encompassing time range from 1,000,000 to 0 generations ago. The runs were evaluated for convergence and were pooled to provide an estimate of current and historical effective population size. Convergence was assessed using the Gelman–Rubin criterion (Gelman and Rubin, 1992) and the test of alternative hypotheses (population decline vs. stable population size) as suggested by Beaumont (1999) was tested using Bayes factors. Calculations and plots were performed in the R statistical programming language (R Development Core Team, 2011) using the packages CODA and ggplot2.

In order to verify reduction in effective population size (*Ne*) or bottleneck effect, we tested for heterozygosity excess in the program BOTTLENECK (Piry et al., 1999) using three different mutation models: the stepwise mutation model, SMM (Ohta and Kimura, 1973); the two-phase model, TPM (Di Rienzo et al., 1994); and the infinite alleles model, IAM (Estoup et al., 1995). Genetic bottlenecks can also leave a signature in the ratio of number alleles to the allele size range (the *M*-ratio), where a bottleneck depletes the number of alleles faster than reducing allelic size range of the microsatellite (Garza and Williamson, 2001). We calculated the *M*-ratio using ARLEQUIN, and considered a reduction in the number of alleles to occur when *M* < 0.68, as suggested by Garza and Williamson (2001).

## Results

### Genetic diversity of *C. macropomum*

**Figure 2:**
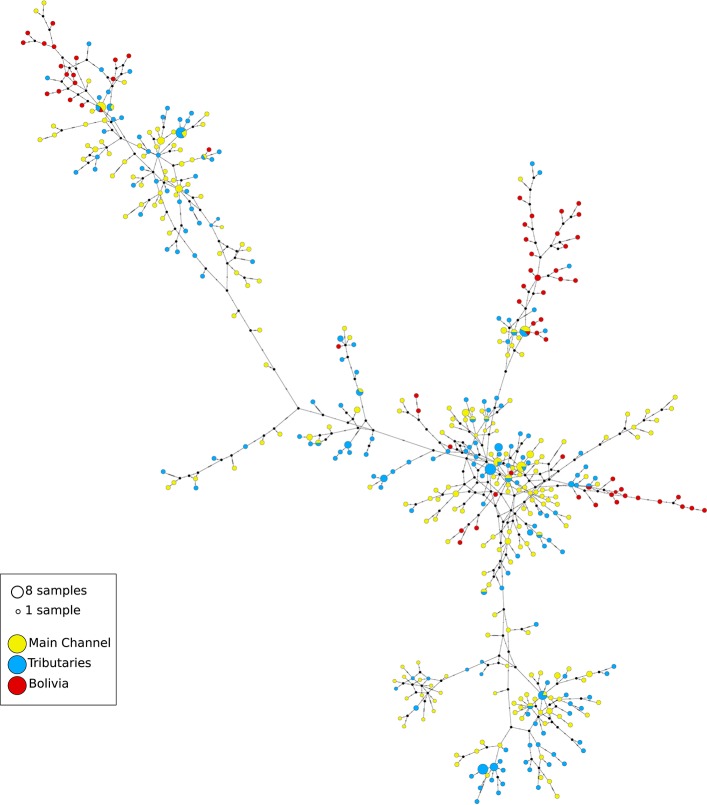
Haplotype network of *Colossoma macropomum* haplotypes estimated using Network. Each line represents a single mutation. Circle size correspond to the number of observations, and missing haplotypes remain unfilled. Colors corresponds to observation of a haplotype in one of three main regions (yellow = mainstream of Amazon River, blue = tributaries of the Amazon River, and red = Bolivian sub-basin).

Eight hundred and thirty-nine base pairs from the control region and 732 base pairs from the ATPase6/8 gene were obtained from 539 individuals. Approximately 5% of the samples were resequenced to confirm the sequences obtained. The sequences of the mitochondrial gene fragments were concatenated, resulting in a total of 1,561 base pairs. A total of 444 haplotypes were found, 400 of which were unique. The haplotype network showed numerous reticulations between haplotypes (Figure 2). There was very little clustering among the haplotypes found at most localities, implying in a high degree of gene exchange. High and relatively homogeneous values of haplotype diversity was found, ranging from *h* = 0.895 in Porto Velho to *h* = 1.000 at nine of the 21 sampled localities (Table 1).

**Table 1:** Main genetic pattern estimates from mtDNA and microsatellites of *C. macropomum* individuals from the Amazon drainage and Bolivian sub-basin.

							**mtDNA**								**Microsatellites**				
**Basin**	**Locality**	**River**	Water type^*^	**N**	***H***	***S***	***h***	**π**	**N**	**A**	**Ana**	**AR**	**PAR**	**AGD**	**Ho-He**	**FIS**	**Ne (Cis)**	**p_SMM**	**Mvalue**
	Mexiana	Amazon	White	15	15	14	1.000 ± 0.024	0.009 ± 0.005	31	139	11.5	6.23	0.12	0.791 ± 0.407	0.779–0.791	**0.049**	4183.1 (140.7-inf)	**0.040**	0.71
	Almeirim	Amazon	White	21	20	14	0.995 ± 0.016	0.009 ± 0.005	36	150	12.5	6.31	0.13	0.781 ± 0.402	0.754–0.781	**0.070**	−234.4 (1094.3-inf)	**0.008**	0.80
	Santarém	Lower Tapajós	Clear	26	26	24	1.000 ± 0.010	0.012 ± 0.006	36	143	11.9	6.29	0.13	0.771 ± 0.397	0.680–0.771	**0.144**	−266.3 (845.1-inf)	0.243	0.78
	Itaituba	Tapajós	Clear	32	24	15	0.977 ± 0.014	0.011 ± 0.006	32	138	11.5	6.01	0.20	0.762 ± 0.393	0.734–0.762	**0.102**	476.9 (102.6-inf)	**0.011**	0.68
	Jacareacanga	Tapajós	Clear	29	19	6	0.958 ± 0.020	0.009 ± 0.005	30	134	11.1	6.04	0.13	0.757 ± 0.391	0.730–0.757	**0.078**	−2610.5 (169.4-inf)	0.277	0.79
	Oriximiá	Lower Trombetas	Clear	34	34	30	1.000 ± 0.007	0.012 ± 0.006	32	139	11.5	6.04	0.12	0.773 ± 0.399	0.715–0.773	**0.102**	275.7 (89.5-inf)	0.118	0.79
	Nhamundá	Lower Nhamundá	Black	22	21	16	0.995 ± 0.015	0.011 ± 0.005	22	127	10.5	6.25	0.19	0.766 ± 0.398	0.727–0.766	**0.078**	100.3 (45.1-inf)	**0.034**	0.74
	Parintins	Amazon	White	20	20	14	1.000 ± 0.015	0.012 ± 0.006	35	148	12.3	6.13	0.17	0.768 ± 0.396	0.719–0.768	**0.081**	96.2 (57.9-233)	**0.012**	0.76
Amazon	Borba	Madeira	White	19	19	13	1.000 ± 0.017	0.013 ± 0.007	23	131	10.9	6.13	0.21	0.776 ± 0.403	0.775–0.776	**0.023**	22.3 (16.4-32.3)	**0.037**	0.74
	Humaitá	Madeira	White	22	20	17	0.991 ± 0.016	0.011 ± 0.005	22	110	9.10	5.66	0.10	0.780 ± 0.405	0.746–0.780	**0.077**	11.6 (8.8-15.6)	**0.042**	0.75
	Porto Velho	Madeira	White	21	14	9	0.895 ± 0.061	0.011 ± 0.006	21	103	8.5	5.59	0.15	0.737 ± 0.385	0.674–0.737	**0.143**	39.7 (24.3-86.4)	0.249	0.64
	Manaus	lower Negro	Black	30	29	22	0.997 ± 0.009	0.012 ± 0.006	35	152	12.6	6.38	0.14	0.785 ± 0.404	0.738–0.785	**0.090**	−437.1 (333.5-inf)	**0.002**	0.77
	Tapauá	Purus	White	28	28	27	1.000 ± 0.009	0.014 ± 0.007	28	144	12.0	6.37	0.18	0.766 ± 0.396	0.755–0.766	**0.056**	−168.7 (73.5-inf)	**0.037**	0.73
	Boca do Acre	Purus	White	21	21	19	1.000 ± 0.014	0.013 ± 0.007	21	130	10.8	6.35	0.27	0.771 ± 0.401	0.742–0.771	**0.076**	240.3 (62.6-inf)	0.112	0.73
	Coari	Lake Coari	Black	20	20	15	1.000 ± 0.015	0.012 ± 0.006	29	141	11.7	6.20	0.16	0.762 ± 0.394	0.683–0.762	**0.123**	924.8 (119.3-inf)	**0.002**	0.78
	Tefé	Lake Tefé	Black	18	16	9	0.986 ± 0.022	0.012 ± 0.006	20	117	9.70	6.04	0.16	0.751 ± 0.392	0.733–0.751	**0.058**	652.6 (86.4-inf)	0.439	0.64
	Carauari	Juruá	White	25	25	25	1.000 ± 0.011	0.013 ± 0.007	25	141	11.6	6.53	0.18	0.790 ± 0.409	0.760–0.790	**0.067**	−448.2 (174.2-inf)	0.244	0.71
	Eirunepé	Juruá	White	16	15	13	0.991 ± 0.025	0.010 ± 0.005	20	113	9.40	6.00	0.08	0.797 ± 0.414	0.754–0.797	**0.066**	47.9 (30.1-100.3)	0.248	0.73
	Fonte Boa	Amazon	White	20	18	10	0.989 ± 0.019	0.011 ± 0.005	31	142	11.8	6.18	0.16	0.773 ± 0.399	0.736–0.773	**0.050**	−309.0 (250.3-inf)	**0.036**	0.73
	Tabatinga	Amazon	White	31	30	24	0.997 ± 0.008	0.013 ± 0.007	37	146	12.1	6.15	0.15	0.779 ± 0.401	0.734–0.779	**0.077**	40.0 (77.0-527.4)	0.290	0.76
Bolivian	Guajará-Mirim	Guaporé	White	69	68	64	0.999 ± 0.002	0.012 ± 0.006	38	123	10.2	5.32	0.09	0.714 ± 0.369	0.677–0.714	**0.087**	−329.8 (443.5-inf)	**0.038**	0.75

N, number of individuals; H, number of haplotypes; S, singletons; h, gene diversity; π, nucleotide diversity; A, number of alleles; A_NA_, average number of alleles per locus; AR, allelic richness; PAR, private allelic richness; AGD, average genetic diversity; H_O_, observed heterozygosity; H_E_, expected heterozygosity; F_IS_, endogamy index; Ne [CI], effective population size [95% confidence intervals], p_SMM = Stepwise Mutation Model from Bottleneck program, Mvalue = M-ratio using ARLEQUIN (Garza and Williamson index). Values in bold are significant at 0.05.

* *Classification of water types based in Sioli (1984) and Venticinque et al. (2016)*.

A total of 604 individuals were genotyped for 13 microsatellite loci. The data revealed no evidence of allelic stutters or large allele dropouts (genotyping errors) and neither linkage disequilibrium (LD). However, deviation from the Hardy-Weinberg equilibrium (EHW) was observed at the Cm1E3 locus in 17 of the 21 sampled localities and the locus was therefore removed from the population analyses. Genetic variability parameters were quite homogeneous among the individuals from different localities (See Table 1). The expected heterozygosity (*H*_*E*_) ranged from 0.714 in Guajará-Mirim to 0.797 in Eirunepé (Juruá River). Mean heterozygosity was 0.777 ± 0.395 for all loci and all locations (Supplementary Table S3). Allelic richness varies from 5.32 alleles in Guajará-Mirim (Guaporé River) to 6.53 in Carauari (Juruá River). The endogamy coefficient (*F*_*IS*_) ranged from 0.023 to 0.144 and was significant for all localities.

### Population structure

AMOVA of the mtDNA data demonstrated that more than 90% of genetic variance was within sampling sites. When AMOVA was performed without Guajará-Mirim (Bolivian sub-basin), Φ_ST_ was 0.032, which is lower than the Φ_ST_ = 0.062 found in the analysis including all sampling sites. Considering the tributaries vs. locations of the main channel, Φ_ST_ was 0.052. Nonetheless, AMOVA was significant for all three datasets analyzed. The result of the Mantel test was non-significan*t* (*r* = 0.1587, *P* = 0.157), demonstrating no correlation between the genetic distances of the sampling sites and their respective geographic distances.

Global AMOVA of microsatellite data resulted in partitioning more than 98% variance within sites (*F*_*ST*_ = 0.0111, *P* = 0.00124). Based in this result, additional AMOVA tests were implemented assuming two main groups: Amazon basin vs. Bolivia basin and, within the Amazon basin, tributaries vs. main channel. The AMOVA results were significant for both analyses (*F*_*ST*_ = 0.0192, *P* = 0.0478; *F*_*ST*_ = 0.0086, *P* = 0.9277; *F*_*ST*_ = 0.0026, *P* = 0.0004), respectively.

**Figure 3:**
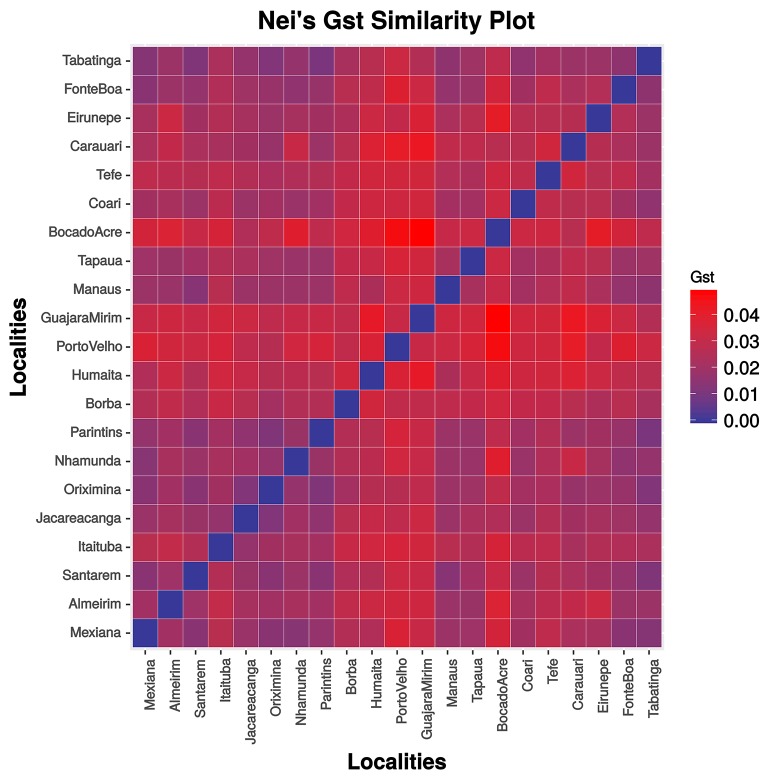
Graphic representation of pairwise Hedrick's *G*_*ST*_ values.

The matrix of pairwise *G*_*ST*_ (Figure 3, Supplementary Table S4) and *F*_*ST*_ values (Supplementary Table S5) were congruent and indicated population structuring. Significant values were observed for almost all comparisons involving the Bolivian sub-basin (Guapore River), and the Madeira (Porto Velho, Humaita, Borba), upper Jurua (Eirunepé), upper Purus (Boca do Acre), and upper Tapajós (Itaituba, Jacareacanga) rivers. The Mantel test was significant (*r* = 0.34260, *P* < 0.05) only when the Bolivian sub-basin was included in the analysis.

**Figure 4:**
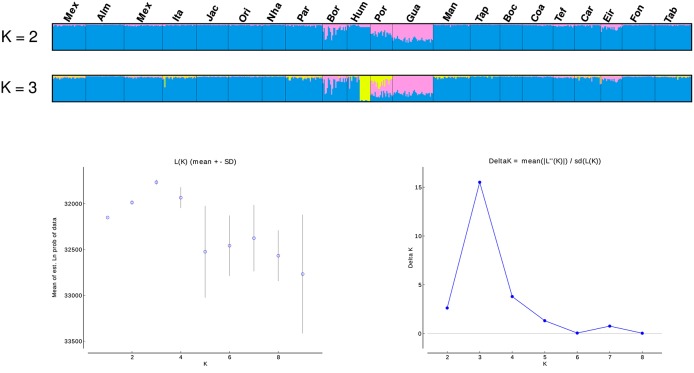
Population structure based on STRUCTURE analysis (*K* = 2, and *K* = 3) of 13 microsatellites. Each vertical bar represents an individual. Estimates of the number of populations (K) based on the mean likelihood Ln (K) and the delta K statistic (Evanno et al., 2005). Locality codes are: Mexiana (Mex), Almeirim (Alm), Santarém (San), Itaituba (Ita), Jacareacanga (Jac), Oriximiná (Ori), Nhamundá (Nha), Parintins (Pin), Borba (Bor), Humaitá (Hum), Porto Velho (Pve), Guajará-Mirim (Gua), Manaus (Mao), Tapauá (Tap), Boca do Acre (Bda), Coari (Coa), Tefé (Tef), Carauari (Car), Eirunepé (Eir), Fonte Boa (Fbo), Tabatinga (Tab).

**Figure 5:**
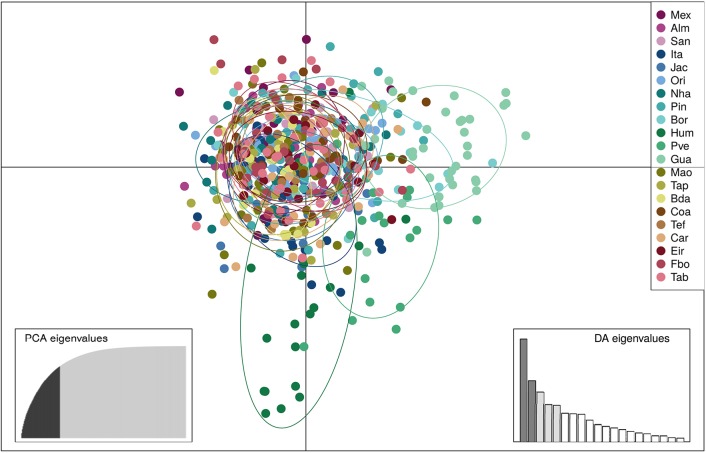
Results of the Discriminant Analysis of Principal Components (DAPC) showing the scatterplot of the first two principal components based on 13 microsatellite loci of 604 individuals of *Colossoma macropomum* from 21 sampling locations. Discriminant function 2 on the x axis and discriminant function 1 on the y axis. In the DAPC graph circles represent different individuals, and colors different sampling localities. Locality codes are: Mexiana (Mex), Almeirim (Alm), Santarém (San), Itaituba (Ita), Jacareacanga (Jac), Oriximiná (Ori), Nhamundá (Nha), Parintins (Pin), Borba (Bor), Humaitá (Hum), Porto Velho (Pve), Guajará-Mirim (Gua), Manaus (Mao), Tapauá (Tap), Boca do Acre (Bda), Coari (Coa), Tefé (Tef), Carauari (Car), Eirunepé (Eir), Fonte Boa (Fbo), Tabatinga (Tab).

SAMOVA analyses indicated the existence of two geographic groups, one group comprised of Guajará-Mirim and another group comprising all remaining localities. At *K* = 2 (Group 1: Guajará-Mirim; Group 2: other sampling sites in the Brazilian Amazon basin), *F*_*CT*_ was maximized for both the mtDNA and microsatellite datasets, but with significant support only for the mtDNA data. Bayesian analyses implemented in STRUCTURE v2.3.4 identified three biological groups. The highest posterior probability was LnP (*K* = 3) = −31766.0000. The three populations comprised individuals from the Bolivian sub-basin and the Brazilian Amazon basin. Individuals from the three localities in the Madeira River showed a linear gradient of admixture between these two populations, and a contribution of an additional biological group principally within the Humaitá locality (Figure 4). Results based on DAPC analysis displayed a general pattern of low genetic differentiation (Figure 5), however, as observed in the previous results, some individuals from upper Madeira River and Guaporé drainage are partially differentiated from the other localities.

### Gene flow

Results of the isolation-with-migration analyses using microsatellite data are in Supplementary Table S6. Thus, Mexiana and Tabatinga (on the Amazon River) were paired, and a group denominated the main channel was formed by randomly sampling 30 individuals from among the sampling sites of the main Amazon River channel, which was then analyzed with upper-most tributary localities: Jacareacanga (Tapajós River), Guajará-Mirim (Bolivian sub-basin), Boca do Acre (Purus River), and Eirunepé (Juruá River) (Table 2). The result indicated bidirectional gene flow between all localities. In all cases, the direction of migration from upstream areas of tributaries to the central Amazon basin predominated except in the case of the Jacareacanga locality.

**Table 2:** Demographic parameters estimated in IMa2 program for microsatellite data.

**Localities**	***Ne*_1_**	***Ne*_2_**	***Ne*_A_**	**T**	***Nm1* (receiving)**	***Nm2* (receiving)**
1-Mexiana and 2-Tabatinga	2,209 (637–26,757)	2,476 (311–21,895)	15,412 (8,287–24,029)	2.5 (0.0–2,000)	11.37 (4.37–1,365)	0.87 (0.87–1,001)
1-Jacareacanga and 2- Main channel	5,068 (1,705–11,114)	2,568 (523–5,932)	16,569 (10,796–25,751)	1,212 (121–6,316)	0.36 (0.36–24.48)	8.65 (1.06–18.71)
1- Guajará-Mirim and 2-Main channel	892 (206–945)	3,959 (892–7,620)	18,695 (12,654–29,405)	725.7 (43–4,892)	4.87 (1.64–8.35)	0.23 (0.13–10.83)
1- Boca do Acre and 2-Main channel	8,630 (3,548–39,410)	2,457 (357–6,152)	17,910 (12,157–29,500)	320.3 (18–6,202)	27.11 (7.87–396.2)	6.33 (0.36–20.72)
1-Eirunepé and 2-Main channel	3,317 (1,283–7,045)	4,237 (1,767–7,917)	19,732 (12,904–33,725)	1,855 (436–7,541)	5.27 (0.47–14.56)	1.11 (0.26–13.12)

*Effective population size Ne = 4 Neμ/4 μx3 (Ne_1_ of the locality1, Ne_2_ of the locality 2 and Ne_A_ of the ancestor of the two localities); current time in years T = (t x μ) × 3; number of migrants per generation Nm = Mθ/2. Values in parentheses = minimum and maximum of confidence limits. Three year generation time is assumed*.

The results of MIGRATE analyses supported substantial levels of geneflow between sites in the main stream of the Amazon, but reduced gene flow levels between localities at tributary headwaters, and of the Madeira River (Table 3). The genetic parameters estimated for *C. macropomu*m in MIGRATE version 3.1.3 inferred from microsatellites data is reported in Supplementary Table S7.

**Table 3:** Demographic parameters estimated in the program Migrate v3.1.3 for microsatellite data.

	**Mex**	**Alm**	**San**	**Ita**	**Jac**	**Ori**	**Nha**	**Pin**	**Bor**	**Hum**	**Pve**	**Gua**	**Mao**	**Tap**	**Bda**	**Coa**	**Tef**	**Car**	**Eir**	**Fbo**	**Tab**
Mex	−	4.2	3.2	1.2	0.5	2.2	3.9	4	1.5	0.5	0.2	0.8	0.3	4.5	1.1	2.7	1.8	1.6	0.9	4.2	13.6
Alm	15.5	—	2.5	0.6	0.5	2.8	5.2	5.3	1.1	0.6	0.3	0.4	1.8	2.7	1.1	3.0	0.8	3.9	0.3	2.6	3.1
San	11.3	2.9	—	1.1	0.3	2.8	3.0	1.5	1.8	0.5	0.1	0.2	1.0	3.1	1.2	4.1	1.2	2.2	0.7	7.0	2.8
Ita	15.9	1.1	2.8	—	0.3	6	1.2	3.6	2.3	0.5	0.7	0.4	0.9	1.6	3.4	4.1	5.7	5.7	0.5	5.9	3.0
Jac	16.4	3.3	4.3	0.8	—	4.3	9.9	2.8	0.3	0.5	0.6	0.2	1.3	10.5	0.4	2.5	5.9	4.0	0.3	2.3	1.9
Ori	10.3	3.9	4	0.7	0.1	—	5.2	2.0	3.6	0.6	0.7	0.4	1.3	3.8	1.1	2.1	1.2	3.0	0.2	4.7	3.3
Nha	23.3	4.9	3.4	0.5	0.4	4.3	—	4.2	3.0	0.3	0.3	0.5	0.8	4.1	0.7	1.7	2.4	2.2	0.1	3.9	1.6
Pin	11.7	2.9	5	0.7	0.1	3.7	4.4	—	4.2	0.7	0.8	0.8	1.0	5.0	1.3	1.8	1.8	5.8	0.3	2.6	1.8
Bor	14.1	1.8	1.9	0.8	0.6	1.2	5.3	3.3	—	1	0.6	0.4	1.6	2.2	1.3	1.8	1.7	9.0	0.2	3.4	1.4
Hum	15.5	8.3	2.4	4.2	0.4	3.5	7.4	1.8	4.0	—	0.1	0.3	2.0	2.2	0.9	4.5	1.8	1.6	0.3	5.9	3.0
Pve	17.3	5.5	0.5	2.1	0.3	2.2	2.0	2.3	2.1	0.6	—	0.4	1.0	5.0	0.5	3.5	3.3	3.7	0.3	6.0	1.9
Gua	16.9	4.5	4	1.6	0.4	3.6	16	2.0	1.7	0.8	0.5	—	1.3	2.7	0.7	4.0	1.5	7.0	0.7	5.7	1.6
Mao	8.5	7.5	2.9	3.5	0.4	3.6	1.2	4.2	6.0	0.5	0.3	0.5	—	4.5	0.5	0.5	3.0	5.5	0.4	3.9	2.2
Tap	6.2	2.9	0.7	0.3	0.1	2.2	3.0	2.8	0.6	0.4	0.6	0.2	1.5	—	2.1	2.0	0.9	5.5	0.5	3.4	2.7
Bda	13.2	2.9	2.6	0.3	0.2	1.8	2.7	2.0	1.6	0.2	0.5	0.2	2.3	3.8	—	1.7	1.2	1.3	0.3	1.8	1.5
Coa	9.5	3.4	5.1	0.7	0.3	5.9	0.7	4.5	1.0	0.4	0.6	0.6	2.0	7.0	0.6	—	2.6	2.2	1.0	5.2	2.2
Tef	14.6	0.8	2.1	0.3	0.2	1.8	8.8	1.1	1.2	0.6	0.3	0.5	0.9	2.2	0.1	1.7	—	4.7	0.3	7.6	2.8
Car	3.0	3.5	5.5	3.0	0.2	3.6	3.9	0.1	1.8	0.3	0.4	0.5	2.6	5.0	0.8	4.8	2.1	—	0.3	4.2	2.0
Eir	12.2	2.1	1.1	0.8	0.3	2.8	7.4	1.5	0.3	1.7	0.6	0.8	0.3	8.9	0.6	1.5	1.8	3.7	—	5.9	0.8
Fbo	27.5	5	4.5	2.0	0.4	3.5	2.7	1.0	1.4	0.2	0.6	0.2	1.6	7.1	1.1	2.9	0.9	4.0	0.3	—	2.2
Tab	5.5	9.4	4.6	0.7	0.1	8.1	5.8	4.5	3.4	0.5	0.6	0.5	3.0	3.4	1.3	2.87	3.2	0.7	0.3	3.6	—

*Number of migrants per generation Nm = Mθ/2. Row localities are sending individuals, while column localities are receiving individuals. Locality codes are: Mexiana (Mex), Almeirim (Alm), Santarém (San), Itaituba (Ita), Jacareacanga (Jac), Oriximiná (Ori), Nhamundá (Nha), Parintins (Pin), Borba (Bor), Humaitá (Hum), Porto Velho (Pve), Guajará-Mirim (Gua), Manaus (Mao), Tapauá (Tap), Boca do Acre (Bda), Coari (Coa), Tefé (Tef), Carauari (Car), Eirunepé (Eir), Fonte Boa (Fbo), Tabatinga (Tab)*.

### Population demography

Using the genetic parameters from IMa2 analyses (Table 2), the coalescent effective population size did not differ substantially for almost all pair of localities examined. As a whole, effective population sizes were of thousands of individuals, with the exception of Guajará-Mirim.

**Figure 6:**
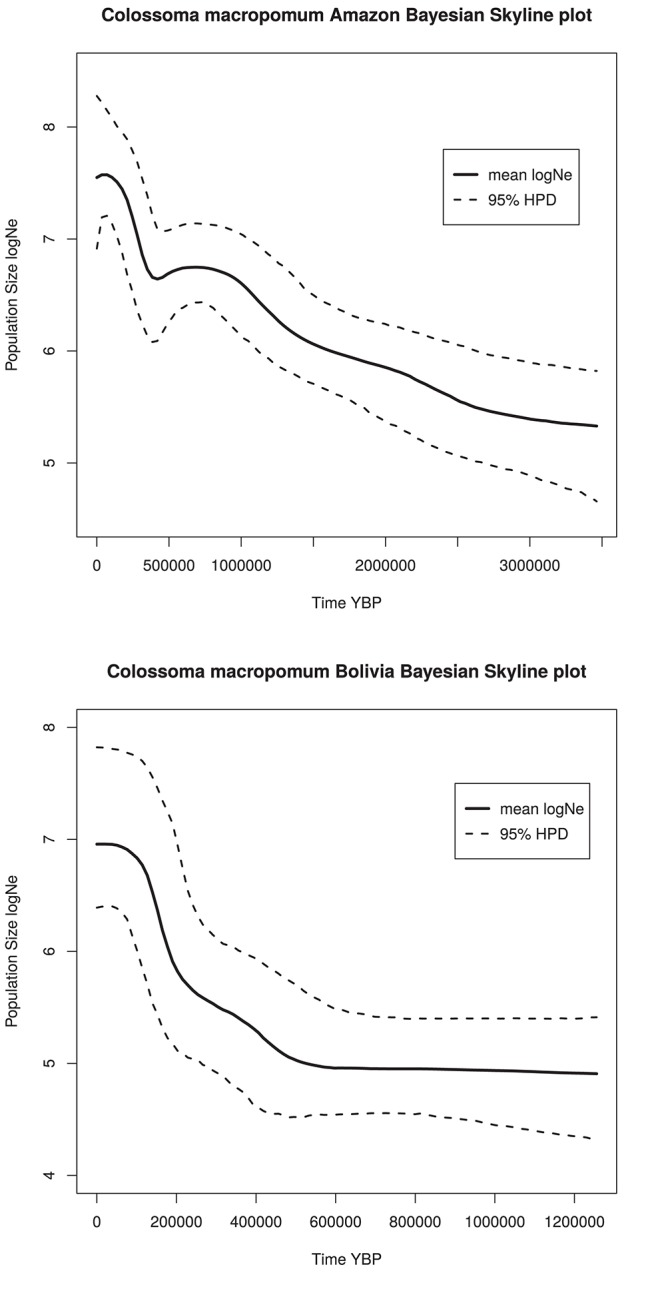
Bayesian skyline plots for Amazon and Bolivian basins.

The Bayesian skyline plot for *C. macropomum* from the Brazilian Amazon demonstrated a strong sign of population expansion, which began slowly approximately 3,000,000 years ago. Demographic growth accelerated considerably approximately 450,000 years ago, with a weak signature of a recent population decline. From the beginning of the initial growth phase, the population size of this species have increased two orders of magnitude from approximately little more than 750 thousand to 75 million individuals in the coalescent history of the populations sampled (Figure 6). Population from Bolivian sub-basin shows demographic growth beginning at 500,000 years ago, with a signature of recent population stability.

Population expansion was also supported by Harpending's raggedness index, which was significantly small (*r* = 0.0046, *P* = 1.0000) considering all samples as well as when considering the two basins separately (Amazon basin: *r* = 0.00060, *P* = 1.0000; Bolivian basin: *r* = 0.0018, *P* = 0.9990) (Table 4). These indexes statistically support the inference of population expansion based on the observation of the distribution of mismatch distribution (Harpending, 1994) for all samples of *C. macropomum*. However, when mismatch distribution was investigated for the basins separately, unimodal distribution was found only within the Amazon basin, whereas multimodal distribution was found for the Bolivian basin (results not shown), which fits a pattern expected under stable population size, although this was not supported by Harpending's raggedness index. The sum of squared deviations (Schneider and Excoffier, 1999) was non-significant for the overall sample (SSD = 0.0020, *P* = 0.6260) as well as the inference performed for the two basins separately (Amazon basin: SSD = 0.0021, *P* = 0.6430; Bolivian basin: SSD = 0.0047, *P* = 0.8420). Thus, these values neither support nor reject the null hypothesis of a demographic population expansion for *C. macropomum* (Table 4). The Tajima's *D* was non-significant for all sampling localities. The same was found for Fu's *Fs*. When considering the basins separately, Fu's *Fs* was significantly negative for the Bolivian basin, suggesting a population expansion.

**Table 4:** Demographic parameters estimated for *Colossoma macropomum*, inferred from mitochondrial DNA data.

			**Mismatch distribution**			**Neutrality test**	
**Basin**	**Localities**	**N**	**Hri (DnaSP)**	**Hri (Arlequin)**	**SSD (Arlequin)**	**Tajima's *D***	**Fu's *Fs***
Brazilian Amazon basin	Mexiana	15	0.0921(*P =* 0.0000)	0.0183 (*P =* 0.6970)	0.0099 (*P =* 0.6630)	−0.3633	−5.6331
	Almeirim	21	0.0908 (*P =* 0.0000)	0.0190 (*P =* 0.3920)	0.0124 (*P =* 0.4090)	−0.4285	−7.4138
	Santarém	26	0.0906 (*P =* 0.0000)	0.0063 (*P =* 0.8370)	0.0041 (*P =* 0.5670)	0.6759	−12.6256^*^
	Itaituba	32	0.0892 (*P =* 0.0000)	0.0119 (*P =* 0.2240)	0.0043 (*P =* 0.5430)	−0.3504	−3.6772
	Jacareacanga	29	0.0882 (*P =* 0.0000)	0.0163 (*P =* 0.4080)	0.0108 (*P =* 0.5590)	−0.0637	−1.2647
	Oriximiná	34	0.0896 (*P =* 0.0000)	0.0021 (*P =* 0.9990)•	0.0031 (*P =* 0.7270)	−0.6243	−19.8803^*^
	Nhamundá	22	0.0885 (*P =* 0.0000)	0.0049 (*P =* 0.9920)•	0.0043 (*P =* 0.8760)	−0.6959	−7.4689
	Parintins	20	0.0921 (*P =* 0.0000)	0.0060 (*P =* 0.9780)•	0.0070 (*P =* 0.6550)	0.0280	−7.7704
	Borba	19	0.0890 (*P =* 0.0000)	0.0105 (*P =* 0.8350)	0.0074 (*P =* 0.7770)	−0.5002	−6.6767
	Humaitá	22	0.0897 (*P =* 0.0000)	0.0061 (*P =* 0.9120)	0.0044 (*P =* 0.8820)	0.1044	−5.4790
	Porto Velho	21	0.0898 (*P =* 0.0000)	0.0251 (*P =* 0.3850)	0.0233 (*P =* 0.3610)	−0.0231	0.3008
	Manaus	30	0.0937 (*P =* 0.0000)	0.0045 (*P =* 0.9690)•	0.0071 (*P =* 0.5700)	−0.0405	−12.9266^*^
	Tapauá	28	0.0881 (*P =* 0.0000)	0.0067 (*P =* 0.7370)	0.0045 (*P =* 0.6940)	−0.4853	−13.0369^*^
	Boca do Acre	21	0.0850 (*P =* 0.0000)	0.0060 (*P =* 0.9340)	0.0075 (*P =* 0.4240)	0.0123	−7.9591
	Coari	20	0.0896 (*P =* 0.0000)	0.0120 (*P =* 0.6820)	0.0665 (*P =* 0.6180)	−0.3213	−7.9575
	Tefé	18	0.0952 (*P =* 0.0000)	0.0298 (*P =* 0.2560)	0.0177 (*P =* 0.2690)	0.0482	−2.7381
	Carauari	25	0.0902 (*P =* 0.0000)	0.0063 (*P =* 0.8510)	0.0034 (*P =* 0.8900)	−0.2137	−10.6621^*^
	Eirunepé	16	0.0921 (*P =* 0.0000)	0.0232 (*P =* 0.6630)	0.0150 (*P =* 0.6460)	−0.3480	−3.6541
	Fonte Boa	20	0.0943 (*P =* 0.0000)	0.0244 (*P =* 0.3590)	0.0264 (*P =* 0.0420)	0.7627	−4.2779
	Tabatinga	31	0.0912 (*P =* 0.0000)	0.0031 (*P =* 0.9690)•	0.0025 (*P =* 0.8240)	−0.2506	−12.8994^*^
Bolivian basin	Guajará–Mirim	69	0.0898 (*P =* 0.0000)	0.0018 (*P =* 0.9990)•	0.0047 (*P =* 0.8420)	0.9462	−24.0981^*^
	All localities	539	0.0912 (*P =* 0.0000)	0.0046 (*P =* 1.0000)•	0.0020 (*P =* 0.6260)	−1.2345	−23.3960

N, number of individuals; Hri, Harpending raggedness index; SSD, sum of square deviations.

Significant values ^*^P < 0.05, ^•^P > 0.95.

**Figure 7:**
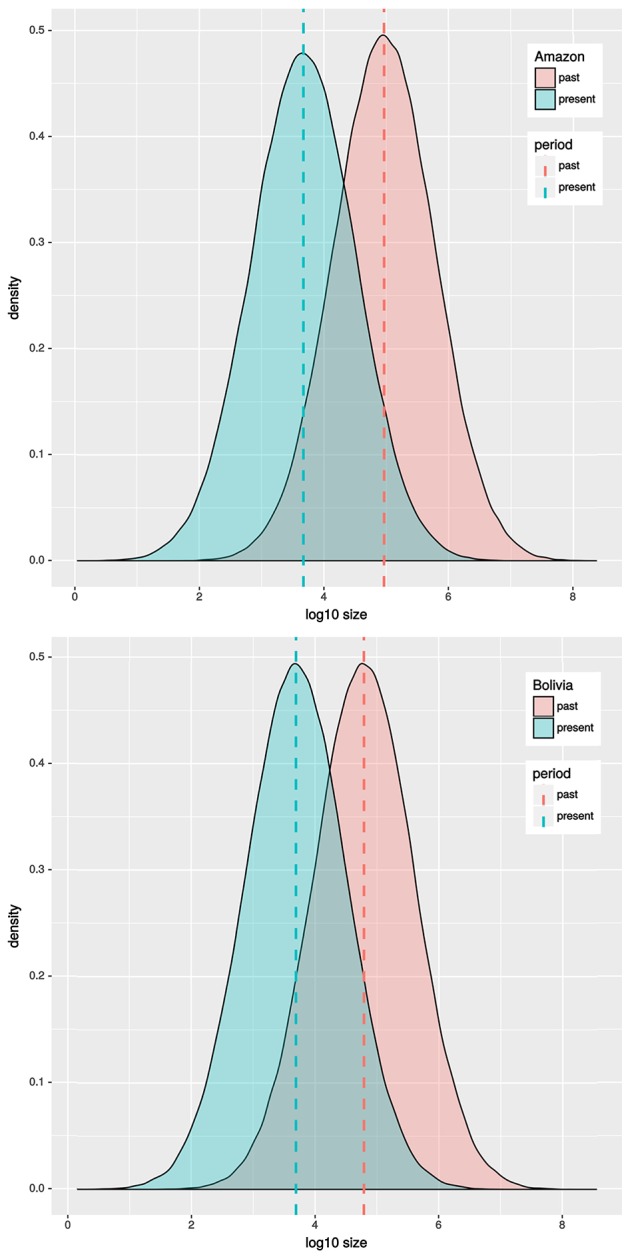
MSVar results for Amazon and Bolivian basins.

Results based in MSVar analyses show that historically *C. macropomum* has undergone a pronounced population decline in both the Amazon and Bolivian basins (Figure 7). The mean estimated ancestral effective population size were at approximately 100,000 individuals, declining recently to approximately 5,000 individuals, an approximately 1.5 orders of magnitude decrease. Demographic decrease was strongly supported (*BF* = 1206) and occurred with 0.9992 probability. Population decline started at approximately 10,000 (Amazon) and 2,500 (Bolivia) years ago. Signs of population reduction in the Bottleneck program were significant for 18 locations under the SMM model, however *M*value showed no signal of population reduction, with exception of individuals from Tefé (Table 1). Effective population sizes (*Ne*) were low for majority of the localities. However, the confidence intervals were also “infinite” for all but the Madeira River, Parintins, Eirunepe and Tabatinga localities.

## Discussion

### The role of rapids in *C. macropomum* gene flow

The use of more variable genetic markers, such as microsatellites, has confirmed some of our earlier findings. *Colossoma macropomum* is not panmictic throughout its distribution area. Considering the entire sample, AMOVA, SAMOVA, STRUCTURE, Φ_ST_ (DNAmt), and *F*_*ST*_/*G*_*ST*_ (microsatellites) analyses, suggested genetic differentiation of the Bolivian sub-basin and Brazilian Amazon basin localities.

Within a given river system, freshwater fishes can either form a large panmictic population or be divided into genetically differentiated groups with sufficient gene flow between groups to maintain the integrity of the meta-population. Gene flow measured indirectly by the number of effective migrants per generation (*Nm*) for DNAmt and microsatellite data evidenced restricted gene flow between the two basins, but enough to maintain the exchange of genes, thereby minimizing effects of genetic drift. The microsatellite data demonstrate that migration between the two basins is bidirectional. The results of both the IMa and MIGRATE analyses show that gene flow is greater from Bolivia to the Brazilian Amazon, which is in agreement with the results described by Farias et al. (2010). The Brazilian part of the Amazon basin receives more migrants, probably through the passive downstream transport of larvae and juveniles.

The genetic differentiation evidenced between the two basins (Brazilian and Bolivian) is associated with the upper Madeira River rapids, which serve as a natural barrier that restricts, but does not prevent, geneflow between the populations of *C. macropomum* of the two basins. The origin of the Bolivian sub-basin is related to the elevation of the Fitzcarrald arch at the beginning of the middle Pliocene (4 to 3 Ma), which gradually isolated the Bolivian basin, resulting in considerable changes in the drainage pattern, in which the main rivers north of Bolivia drain into the Amazon River through the Madeira River (Hoorn et al., 1995; Campbell et al., 2001), which is the largest tributary of the southern margin of the Solimões-Amazonas basin (Lundberg et al., 1998). The Bolivian sub-basin includes the main Beni and Mamoré rivers, as well as approximately 60% of the entire drainage area of the Madeira River (upper part of Madeira) and is separated from the Amazon basin by a set of 18 rapids and cataracts located between Guajará-Mirim and Porto Velho (Cella-Ribeiro et al., 2013). The largest of these cataracts, the Teotônio cataract, constituted the greatest barrier to navigation on this river, as well as to the movement of many species of fish (Goulding et al., 2003). The rapids of the upper Madeira River play an important role in the structuring of populations of other Amazonian aquatic species, such as the river turtle *Podocnemis expansa* (Pearse et al., 2006), river dolphins (Gravena et al., 2014, 2015), the black Amazonian flanelmouth characin *Prochilodus nigricans* (Machado et al., 2017), and the catfish *Brachyplatystoma rousseauxii* (Batista, 2010; Carvajal-Vallejos et al., 2014). The Teotônio cataract, as well as the Jirau cataract, the second largest of the Madeira River, have been submerged by hydroelectric reservoirs.

### Population genetic structure in the amazonas river

The lack of genetic differentiation of *C. macropomum* in the main channel of Amazonas River in the Brazilian Amazon basin was supported by all population structure analyses based on mitochondrial genes and microsatellites loci. These findings confirm the pattern reported by Santos et al. (2007) and Farias et al. (2010) who used mtDNA only (supported by Φ_ST)_), and worked at much smaller geographic scales. However, Φ_ST_ comparisons involving the tributaries were significant even after Bonferroni corrections. Fisher's exact test and Hedrick's *G*_*ST*_ analysis with microsatellites markers show a weak population differentiation between localities, and stronger differentiation involving comparisons with tributaries, and also black water sites. Geneflow between the main stream localities and tributary headwaters and black water sites generally is smaller than one effective migrant per generation which also confirms the migration patterns observed in IMa and MIGRATE analyses. Contrary to this pattern, STRUCTURE analysis shows population differentiation only for upper Madeira (localities below the rapids). STRUCTURE program uses a Bayesian approach to investigate the number of biological groups in the dataset. The discordance, between these analyses, could be due to the fact that *G*_*ST*_ analyses are based on variance in allelic frequencies between/among groups and Fisher's exact test uses contingency tables to test null hypothesis that the alleles are drawn from the same distribution in all populations. These two analyses are more sensitive to detecting smaller and finer levels of genetic differentiation, while STRUCTURE tests for shared system of mating among individuals of the same group. The algorithm of STRUCTURE will not necessarily detect weak structure (Evanno et al., 2005). The population structure observed in *C. macropomum* falls within the category of weak to moderate level of population differentiation (according to Wright, 1965), which could be limiting the sensitivity of the analysis to find more refined substructuring.

Putman and Carbone (2014) emphasize that analyzes used to infer population differentiation have limitations in detecting or not the population structure. Therefore, being conservative for purposes of management and conservation of this species, we consider that the populations of the tributaries (Juruá, Purus, Madeira, Tapajós rivers, and blackwater localities) are different management units until proven otherwise. In this case, the management of fisheries, and seasonal fishing closures during reproductive period, must be effectively respected to preserve the evolutionary potential for the species sustainability. Encouraging aquaculture of the species could also minimize the impact of harvesting natural stocks, which is in fact already occurring.

On the other hand, in the great corridor of the main channel of the Amazon River, from Mexiana (1) to Tabatinga (20) there seem to be not even a signal of isolation-by-distance between the two localities separated by approximately 2,500 Km. The observed lack of genetic structuring of *C. macropomum* in the main channel of the Amazon River basin is probably the result of living in a floodplain environment. The life cycle of this species is tied directly to the seasonal flood cycle of the Amazon; during the flood *C. macropomum* disperses to reproduce and feed in the floodplains and in the flooded forest, while in the dry period fishes become concentrated in lakes and rivers (Araújo-Lima and Goulding, 1998). During the reproduction season, the eggs and larvae are passively transported by the millions to the floodplains, as this species is highly fecund (Araújo-Lima and Goulding, 1998; Araújo-Lima and Ruffino, 2004). This dynamic together with the interlinking of river channels during from flood pulses thus is the primary factor in homogenizing differences among populations of *C. macropomum* (Junk, 1997) and this is observed in molecular data as well. The lack of population structuring is not a unique feature of *C. macropomum*; numerous other species occupying the Amazonian floodplain [e.g., *Prochilodus nigricans* (Machado et al., 2017), *Brycon amazonicus* (Oliveira et al., 2018), and *Paratrygon aiereba* (Frederico et al., 2012)] show this pattern as well. Thus, the pattern found in the present study likely stems from events acting at a macro time scale that affected the region, which, together with current water cycles and the migratory movements of *C. macropomum*, may maintain intra-population homogeneity over generations.

### Genetic diversity: large or small?

Considering that *H*_*O*_ suffers from sampling effects, we used *H*_*E*_ as a minimally biased estimate of diversity (Frankham et al., 2002). The *H*_*E*_ for *C. macropomum* for the microsatellite data were moderate and uniform across the sampling localities, ranging from 0.71 to 0.79 with an average of 0.78. For all microsatellite data, variability measures, such as expected heterozygosity and allelic diversity, were similar to those observed in other exploited migratory Amazonian fishes (Carvajal-Vallejos et al., 2014; Ochoa et al., 2015; Oliveira et al., 2018). A compilation of average *H*_*E*_ values for microsatelite loci obtained from the literature for exploited Amazonian migratory fish shows average *H*_*E*_ values ranging from 0.50 of *Brachyplatystoma platynemum* (Ochoa et al., 2015), to 0.87 of *Semaprochilodus insignis* (Passos et al., 2010). Between this minimum and maximum, one can observe *H*_*E*_ = 0.61 of *Brachyplatystoma rousseauxii* (Batista, 2010), *H*_*E*_ = 0.75 of *Brachyplatystoma vaillantii* (Rodrigues et al., 2009), and *H*_*E*_ = 0.83 of *Brycon amazonicus* (Oliveira et al., 2018). An average of these values (mean *H*_*E*_ = 0.72) is not in agreement with DeWoody and Avise (2000), who report an average *H*_*E*_ = 0.54 for freshwater fishes. The *H*_*E*_ = 0.78 of *C. macropomum* and most of the *H*_*E*_ reported for exploited Amazonian migratory fishes are more similar to the *H*_*E*_ of marine fishes reported by DeWoody and Avise (2000) with a mean *H*_*E*_ = 0.77. In the review of DeWoody and Avise (2000), the authors summarized microsatelite data from North American and European freshwater species, that in comparison to the Amazon basin are geographically very restricted. At 5.5 million km^2^, the Amazon basin is by far the largest hydrographic basin on the planet, and the size of area occupied by many fish species is comparable to that for marine fishes. The Amazon is in a sense a “sea” that provides an expansive and effectively continuous environment for migratory freshwater fishes such as *C. macropomum* and the other cited species. Migratory fishes, whether freshwater or marine, are usually *r* strategists, that is, they have high dispersal capacity, produce a lot of offspring, and in general have a large effective population size, which is turn is reflected in high heterozigosity levels.

In this respect, when compared to freshwater fishes of Europe and North America, the heterozygosities of Amazonian fishes may appear to be high, but this is an illusion. The expected heterozygosities are *on par* with those expected for fishes occupying large areas and having large census numbers. Within the Amazonian species analyzed, the large predatory catfishes of the genus *Brachyplatystoma* have lower *H*_*E*_ as would be expected by their smaller census sizes. At the opposite end of the spectrum are the relatively small, detrivorous and frigivorous migratory characids (*Semaprochilodus, Prochilodus*, and *Brycon*) which have higher *H*_*E*_ as would be expected by their much larger census sizes. *Colossoma macropomum* has an intermediate *H*_*E*_, again a reflection of its frugivorous lifestyle combined with large body size, and thus smaller census size than the other migratory characids but larger census size than the predatory catfishes.

### Population genetic demography

An analysis of historical demography of *C. macropomum* suggested population expansion in the Amazon basin, which is the same scenario suggested by Farias et al. (2010). However, the result of the current study suggest a population size reduction in the Holocene (Figure 5). This result is confirmed by the very recent population decline observed in the Skyline plot analyses (Figure 4). Similar pattern are observed for the Bolivian basin, however, very recent population decline is not evidenced in the Skyline plot.

Most studies conducted in the Amazon involving fish species such as *Prochilodus nigricans* (Machado et al., 2017), *Brachyplatystoma rousseauxii* (Batista and Alves-Gomes, 2006; Carvajal-Vallejos et al., 2014), and *Brachyplatystoma platynemum* (Ochoa et al., 2015) indicate recent population expansion, at least as indicated by Fu's *Fs* test. The difference in effective population size of *C. macropomum* prior estimated for the Bolivian and Amazon basin broadly corresponded to the relative proportion of potential habitat in the Bolivian basin. This basin accounts for approximately 20% of the total Amazon basin, suggesting that the Bolivian basin had a *C. macropomum* population approximately 20% smaller than the rest of the Amazon basin during the Holocene.

Glacial and inter-glacial periods of the Pleistocene exerted considerable impact on the climate, which consequently affected the vegetation in South America (Ledru et al., 1996) and also had impact on aquatic and terrestrial fauna. It is during the Late Pleistocene that *C. macropomum* in the Amazon basin began expanding (Figure 4) associated with the expansion of the várzea-like habitat (Irion and Kalliola, 2010). Therefore, the population expansion of *C. macropomum* in the Amazon basin likely occurred due to the increase in the availability of habitat for this species starting in the later half of the Pleistocene; however, population growth is no longer observed in the Holocene.

Corroborating the observation for the Holocene, analyses of microsatellite data in MSVar indicate that *C. macropomum* has undergone a pronounced population decline in both drainages during the Holocene (10,000 years ago—Amazon and 2,500 years ago Bolivia), probably due to climate change related to Last Glacial Maximum and during the mid-Holocene epoch (Wang et al., 2017). Demographic decrease was strongly supported (*BF* = 1206) and occurred with 0.9992 probability.

The demographic decrease was from approximately 100,000 effective individuals to approximately 5,000 individuals, an approximate 1.5 orders of magnitude decrease. Similar values for current effective population size were inferred using IMa2 (Table 2). By any measure, the effective population size is small, and much smaller than in the last several thousand years.

DeWoody and Avise (2000) estimate that at equilibrium *H*_*E*_ = 0.79 represents 25,000 effective individuals assuming a substitution rate of 10^−4^. Our estimate of substitution rate from MSVar was 10^−3.81^, or just about 17,000 effective individuals are expected at equilibrium. In this sense, the *Ne* values of *C. macropomum* are below an equilibrium expectation, which also suggests a current reduction of *Ne*. In fact, the results of the Bottleneck program indicate decrease in population size in the majority of sampling localities, which is also corroborated by the *Ne* values (Table 1). The only major event that may have contributed to population decrease of *C. macropomum* in the nowadays, within a time window of decades, is the over-exploitation of the species and the destruction of its floodplain habitat. Natural stocks of the *C. macropomum* suffer from overfishing and juveniles currently account for the largest part of the catch (Barthem and Goulding, 2007). Although aquaculture of *C. macropomum* has grown in recent years, there is strong evidence that the natural population of this species are still depressed because of over-fishing. This can be evidenced in the continuous reduction of the tonnage landed in the port of Manaus and other major Amazonian ports. Araújo-Lima (2002) report that in 1976 *C. macropomum* reached 16,000 tons/year landed in the port of Manaus, while data from late 1990's indicated less than 4 thousand tons. During this time, the population of Manaus more than doubled. Furthermore, another worrying factor is the mean size of the fish landed, with juveniles representing the majority of the catch (Barthem and Goulding, 2007). The average size of the fish landed in the main markets in the Amazon suggests that most individuals are fished before reaching sexual maturation, which in the case of females occurs between 50 and 55 cm (Isaac and Ruffino, 2000).

In conclusion, naturally exogamous species with large census sizes have considerable genetic diversity and large effective population sizes (Frankham et al., 2002). In this context, *C. macropomum* has levels of genetic diversity that are *on par* with expectations for species of similar lifestyle and body size. However, with the historical decline of *C. macropomum* populations, it is evident that part of the genetic diversity that existed in the past has been lost. Still the remaining diversity is representative of this species's historical genetic diversity and it is this genetic diversity that can secure the recovery and long-term persistence of natural populations of *C. macropomum* in the Amazon basin.

## Ethics statement

All field collections were authorized by IBAMA/SISBIO 11325-1, and access to genetic resources was authorized by permit No. 034/2005/IBAMA. Field collection permits are conditional that collection of organisms be undertaken in accordance with the ethical recommendations of the Conselho Federal de Biologia (CFBio; Federal Council of Biologists), Resolution 301 (December 8, 2012).

## Author contributions

IF and TH conceived the experiment and obtained funding. MS, IF, and TH conducted fieldwork, collected specimens, analyzed the results, and wrote the manuscript. MS collected molecular data. All authors contributed to and reviewed the manuscript.

### Conflict of interest statement

The authors declare that the research was conducted in the absence of any commercial or financial relationships that could be construed as a potential conflict of interest.

## References

[B1] Araújo-Lima C. A. R. M. (2002). Piscicultura extensiva de tambaqui na floresta de várzea, in Livro de Resultados dos Projetos de Pesquisa Dirigida (PPDS)-PPG7 (Brasília: MCT), 131–135.

[B2] Araújo-Lima C. A. R. M. Goulding M. (1998). Os Frutos do Tambaqui. Ecologia, Conservação e Cultivo na Amazônia. Belem: Sociedade Civil Mamirauá-MCT-CNPq.

[B3] Araújo-Lima C. A. R. M. Ruffino M. L. (2004). Migratory fishes of the Brazilian Amazon, in Migratory Fishes of South America. Biology, Fisheries, and Conservation Status, eds Carolsfield J. Harvey B. Ross C. Baer A. (Victoria, BC: Co-published by World Fisheries Trust/World Bank/International Development Research Center), 233–302.

[B4] Barthem R. B. Fabré N. N. (2003). Biologia e diversidade dos recursos pesqueiros da Amazônia, in A Pesca e os Recursos Pesqueiros na Amazônia Brasileira, ed Ruffino M. L. (Manaus, AM: ProVarzea), 11–55.

[B5] Barthem R. B. Goulding M. (2007). An Unexpected Ecosystem: The Amazon as Revealed by Fisheries. St. Louis, MO: Amazon Conservation Association and Missouri Botanical Garden Press.

[B6] Batista J. S. (2010). Caracterização Genética da Dourada-Brachyplatystoma Rousseauxii, Castelnau, 1855 (Siluriformes - Pimelodidae) na Amazônia por Meio de Marcadores Moleculares Mitocondriais e Microssatélites: Subsídios Para Conservação e Manejo. Tese de Mestrado do Programa de Pós-graduação de Genética, Conservação e Biologia Evolutiva, Instituto Nacional de Pesquisas da Amazônia, Manaus, Brasil.

[B7] Batista J. S. Alves-Gomes J. A. (2006). Phylogeography of *Brachyplatystoma rousseauxii* (Siluriformes - Pimelodidae) in the Amazon Basin offers preliminary evidence for the first case of “homing” for an Amazonian migratory catfish. Gen. Mol. Res. 5, 723–740. 17183483

[B8] Beaumont M. A. (1999). Detecting population expansion and decline using microsatellites. Genetics 153, 2013–2029. 10581303 10.1093/genetics/153.4.2013 PMC1460853

[B9] Beerli P. Felsenstein J. (2001). Maximum likelihood estimation of a migration matrix and effective population sizes in n subpopulations by using a coalescent approach. Proc. Natl. Acad. Sci. U.S.A. 98, 4563–4568. 10.1073/pnas.081068098 11287657 PMC31874

[B10] Campbell K. E. Jr. Heizler M. Frailey C. D. Romero Pittman L. Prothero D. R. (2001). Upper Cenozoic chronostratigraphy of the southwestern Amazon Basin. Geology 29, 595–598. 10.1130/0091-7613(2001)029<0595:UCCOTS>2.0.CO;2

[B11] Carvajal-Vallejos F. M. Duponchelle F. Desmarais E. Cerqueira F. Querouil S. Nuñez J.. (2014). Genetic structure in the Amazonian catfish *Brachyplatystoma rousseauxii*: influence of life history strategies. Genetica 142, 323–336. 10.1007/s10709-014-9777-2 25038864

[B12] Cella-Ribeiro A. Torrente-Vilara G. Hungria D. B. Oliveira M. de (2013). As corredeiras do Rio Madeira, in Peixes do Rio Madeira, eds de Queiroz L. J. Torrente-Vilara G. Ohara W. M. da T. H. Pires S. Zuanon J. Doria C. R. C. (Santo Antonio Energia), 47–53.

[B13] Corander J. Tang J. (2007). Bayesian analysis of population structure based on linked molecular information. Math. Biosci. 205, 19–31. 10.1016/j.mbs.2006.09.015 17087977

[B14] DeWoody J. A. Avise J. C. (2000). Microsatellite variation in marine, freshwater and anadromus fishes compared with other animals. J. Fish Biol. 56, 461–473. 10.1006/jfbi.1999.1210

[B15] DeWoody J. A. Schupp J. Kenefic L. Busch J. Murfitt L. Keim P. (2004). Universal method for producing ROX-labeled size standards suitable for automated genotyping. BioTechniques 37, 348–352. 10.2144/04373BM02 15470886

[B16] Di Rienzo A. Peterson A. C. Garza J. C. Valdes A. M. Slatkin M. Freimer N. B. (1994). Mutational processes of simple-sequence repeat loci in human populations. Proc. Natl. Acad. Sci. U.S.A. 91, 3166–3170. 10.1073/pnas.91.8.3166 8159720 PMC43536

[B17] Do C. Waples R. S. Peel D. Macbeth G. M. Tillett B. J. Ovenden J. R. (2014). NeEstimator v2: re-implementation of software for the estimation of contemporary effective population size (Ne) from genetic data. Mol. Ecol. Res. 14, 209–214. 10.1111/1755-0998.12157 23992227

[B18] Drummond A. J. Rambaut A. (2007). BEAST: bayesian evolutionary analysis by sampling trees. BMC Evol. Biol. 7:214. 10.1186/1471-2148-7-214 17996036 PMC2247476

[B19] Dupanloup I. Schneider S. Excoffier L. (2002). A simulated annealing approach to define the genetic structure of populations. Mol. Ecol. 11, 2571–2581. 10.1046/j.1365-294X.2002.01650.x 12453240

[B20] Estoup A. Tailliez C. Cornuet J.-M. Solignac M. (1995). Size homoplasy and mutational processes of interrupted microsatellite in two bee species, *Apis mellifera* and *Bombus terrestris* (Apidae). Mol. Biol. Evol. 12, 1074–1084. 8524041 10.1093/oxfordjournals.molbev.a040282

[B21] Evanno G. Regnaut S. Goudet J. (2005). Detecting the number of clusters of individuals using the software STRUCTURE: a simulation study. Mol. Ecol. 14, 2611–2620. 10.1111/j.1365-294X.2005.02553.x 15969739

[B22] Excoffier L. Lischer H. E. L. (2010). Arlequin suite ver 3.5: a new series of programs to perform population genetics analyses under Linux and Windows. Mol. Ecol. Resour. 10, 564–567. 10.1111/j.1755-0998.2010.02847.x 21565059

[B23] Excoffier L. Smouse P. E. Quattro J. M. (1992). Analysis of molecular variance inferred from metric distances among DNA haplotypes: application to human mitochondrial DNA restriction data. Genetics 131, 479–491. 1644282 10.1093/genetics/131.2.479 PMC1205020

[B24] Farias I. P. Torrico J. P. García-Dávila C. da Santos M. C. F. Hrbek T. Renno J.-F. (2010). Are rapids a barrier for floodplain fishes of the Amazon basin? A demographic study of the keystone floodplain species *Colossoma macropomum* (Teleostei: Characiformes). Mol. Phylogenet. Evol. 56, 1129–1135. 10.1016/j.ympev.2010.03.028 20362063

[B25] Frankham R. Ballou J. D. Briscoe D. A. (2002). Introduction to Conservation Genetics. Cambridge: Cambridge University Press.

[B26] Frederico R. G. Farias I. P. Araújo M. L. G. Charvet-Almeida P. Alves-Gomes J. A. (2012). Phylogeography and conservation genetics of the Amazonian freshwater stingray *Paratrygon aiereba* Müller & Henle, 1841 (Chondrichthyes: Potamotrygonidae). Neotrop. Ichthyol. 10, 71–80. 10.1590/S1679-62252012000100007

[B27] Fu Y.-X. (1997). Statistical tests of neutrality of mutations against population growth, hitchhiking and background selection. Genetics 147, 915–925. 9335623 10.1093/genetics/147.2.915 PMC1208208

[B28] Garza J. C. Williamson E. G. (2001). Detection of reduction in population size using data from microsatellite loci. Mol. Ecol. 10, 305–318. 10.1046/j.1365-294X.2001.01190.x 11298947

[B29] Gelman A. Rubin D. B. (1992). Inference from iterative simulation using multiple sequences. Stat. Sci. 7, 457–472. 10.1214/ss/1177011136

[B30] Goulding M. Barthem R. B. Ferreira E. J. G. (2003). The Smithsonian Atlas of the Amazon. Washington, DC: Smithsonian Institution Press.

[B31] Goulding M. Carvalho M. L. (1982). Life history and management of the tambaqui (*Colossoma macropomum*, Characidae): an important amazonian food fish. Rev. Bras. Zool. 1, 107–133. 10.1590/S0101-81751982000200001

[B32] Gravena W. da Silva V. M. F. da Silva M. N. F. Farias I. P. Hrbek T. (2015). Living between rapids: genetic structure and hybridization in botos (Cetacea: Iniidae: Inia spp.) of the Madeira River, Brazil. Biol. J. Linn. Soc. 114, 764–777. 10.1111/bij.12463

[B33] Gravena W. Farias I. P. da Silva M. N. F. da Silva V. M. F. Hrbek T. (2014). Looking to the past and the future: were the Madeira River rapids a geographic barrier to the boto (Cetacea: Iniidae)? Conserv. Genet. 15, 619–629. 10.1007/s10592-014-0565-4

[B34] Hall T. (1999). BioEdit: a user-friendly biological sequence alignment editor and analysis program for Windows 95/98/NT. Nucleic Acids Symp. Ser. 41, 95–98. Available online at: http://www.mbio.ncsu.edu/BioEdit/bioedit.html

[B35] Harpending H. C. (1994). Signature of ancient population growth in a low-resolution mitochondrial DNA mismatch distribution. Hum. Biol. 66, 591–600. 8088750

[B36] Hasegawa M. Kishino H. Yano T. A. (1985). Dating of the human-ape splitting by a molecular clock of mitochondrial DNA. J. Mol. Evol. 22, 160–174. 10.1007/BF02101694 3934395

[B37] Hedrick P. W. (2005). A standardized genetic differentiation measure. Evolution 59, 1633–1638. 10.1111/j.0014-3820.2005.tb01814.x 16329237

[B38] Hey J. Nielsen R. (2007). Integration within the Felsenstein equation for improved Markov chain Monte Carlo methods in population genetics. Proc. Natl. Acad. Sci. U.S.A. 104, 2785–2790. 10.1073/pnas.0611164104 17301231 PMC1815259

[B39] Hoorn C. Guerrero J. Sarmiento G. A. Lorente M. A. (1995). Andean tectonics as a cause for changing drainage patterns in Miocene northern South America. Geology 23, 237–240. 10.1130/0091-7613(1995)023<0237:ATAACF>2.3.CO;2

[B40] Hoorn C. Wesselingh F. P. ter Steege H. Bermudez M. A. Mora A. Sevink J.. (2010). Change, landscape evolution, and biodiversity Amazonia through time: Andean uplift, climate. Science 330, 927–931. 10.1126/science.1194585 21071659

[B41] Irion G. Kalliola R. J. (2010). Long-term landscape development processes in Amazonia, in Amazonia: Landscape and Species Evolution: A Look into the Past, eds Hoorn C. Wesselingh F. P. (Chichester: Wiley-Blackwell), 185–197.

[B42] Isaac V. J. Milstein A. Ruffino M. L. (1996). A pesca artesanal no baixo Amazonas: análise multivariada da captura por espécies. Acta Amazon. 26, 185–208. 10.1590/1809-43921996263208

[B43] Isaac V. J. Ruffino M. L. (1996). Population dynamics of tambaqui, *Colossoma macropomum* Cuvier, in the lower Amazon, Brazil. Fish. Manag. Ecol. 1996, 315–333. 10.1046/j.1365-2400.1996.d01-154.x

[B44] Isaac V. J. Ruffino M. L. (2000). Biologia pesqueira do tambaqui, Colossoma macropomum, no Baixo Amazonas, in Recursos Pesqueiros do Médio Amazonas: Biologia e Estatística Pesqueira (Brasília: Edições IBAMA; Coleção meio ambiente; Série Estudos Pesca), 65–88.

[B45] Jombart T. (2008). Adegenet: a R package for the multivariate analysis of genetic markers. Bioinformatics 24, 1403–1405. 10.1093/bioinformatics/btn129 18397895

[B46] Jombart T. Devillard S. Balloux F. (2010). Discriminant analysis of principal components: a new method for the analysis of genetically structured populations. BMC Genet. 11:94. 10.1186/1471-2156-11-94 20950446 PMC2973851

[B47] Junk W. J. (1997). The Central Amazon System: Ecology of a Pulsing System. Berlin: Springer Verlag.

[B48] Kalinowski S. T. (2005). Hp-Rare 1.0: a computer program for performing rarefaction on measures of allelic richness. Mol. Ecol. Notes 5, 187–189. 10.1111/j.1471-8286.2004.00845.x

[B49] Kitada S. Kitakado T. Kishino H. (2007). Empirical bayes inference of pairwise F_ST_ and its distribution in the genome. Genetics 177, 861–873. 10.1534/genetics.107.077263 17660541 PMC2034649

[B50] Kitada S. Nakamichi R. Kishino H. (2017). The empirical Bayes estimators of fine-scale population structure in high gene flow species. Mol. Ecol. Resour. 17, 1210–1222. 10.1111/1755-0998.12663 28241394

[B51] Leberg P. L. (2002). Estimating allelic richness: effects of sample size and bottlenecks. Mol. Ecol. 11, 2445–2449. 10.1046/j.1365-294X.2002.01612.x 12406254

[B52] Ledru M.-P. Braga P. I. S. Soubiès F. Fournier M. Martin L. Suguio K. (1996). The last 50,000 years in the Neotropics (Southern Brazil): evolution of vegetation and climate. Palaeogeogr. Palaeoclimatol. Palaeoecol. 123, 239–257. Available online at: http://www.sciencedirect.com/science/article/B6V6R-3VVT2P4-D/2/b91b6aa40adb8b4b114c150b06404376. 10.1016/0031-0182(96)00105-8

[B53] Librado P. Rozas J. (2009). DnaSP v5: a software for comprehensive analysis of DNA polymorphism data. Bioinformatics 25, 1451–1452. 10.1093/bioinformatics/btp187 19346325

[B54] Lovejoy N. R. Willis S. C. Albert J. S. (2010). Molecular signatures of Neogene biogeographical events in the Amazon fish fauna, in Amazonia: Landscape and Species Evolution: A Look into the Past, eds Hoorn C. Wesselingh F. P. (Oxford: Wiley-Blackwell), 405–417.

[B55] Lundberg J. G. Marshall L. G. Guerrero J. Horton B. Malabarba M. C. S. L. Wesselingh F. P. (1998). The stage for Neotropical fish diversification: a history of tropical South American rivers, in Phylogeny and Classification of Neotropical Fishes, eds Malabarba L. R. Reis R. E. Vari R. P. Lucena Z. M. S. Lucena C. A. S. (Porto Alegre: EDIPUCRS), 13–48.

[B56] Machado V. N. Willis S. C. Hrbek T. Farias I. P. (2017). Population genetic structure of the Amazonian black flannelmouth characin (Characiformes, Prochilodontidae: Prochilodus nigricans Spix and Agassiz, 1829): contemporary and historical gene flow of a migratory and abundant fishery species. Environ. Biol. Fishes 100, 1–16. 10.1007/s10641-016-0547-0

[B57] Mantel N. (1967). The detection of disease clustering and a generalized regression approach. Cancer Res. 27, 209–220. 6018555

[B58] Ochoa L. E. Pereira L. H. G. Costa-Silva G. J. Roxo F. F. da Batista J. S. Formiga K.. (2015). Genetic structure and historical diversification of catfish Brachyplatystoma platynemum (Siluriformes: Pimelodidae) in the Amazon basin with implications for its conservation. Ecol. Evol. 5, 2005–2020. 10.1002/ece3.1486 26045952 PMC4449755

[B59] Ohta T. Kimura M. (1973). A model of mutation appropriate to estimate the number of electrophoretically detectable alleles in a finite population. Gen. Res. 22, 201–204. 10.1017/S0016672300012994 4777279

[B60] Oliveira R. C. Santos M. C. F. Bernardino G. Hrbek T. Farias I. P. (2018). From river to farm: an evaluation of genetic diversity in wild and aquaculture stocks of Brycon amazonicus (Spix & Agassiz, 1829), Characidae, Bryconinae. Hydrobiologia 805, 75–88. 10.1007/s10750-017-3278-0

[B61] Palstra F. P. Fraser D. J. (2012). Effective/census population size ratio estimation: a compendium and appraisal. Ecol. Evol. 2, 2357–2365. 10.1002/ece3.329 23139893 PMC3488685

[B62] Passos K. B. Leão A. S. A. Oliveira D. P. Farias I. P. Hrbek T. (2010). Polymorphic microsatellite markers for the overexploited Amazonian fish, Semaprochilodus insignis (Jardine and Schomburgk 1841). Conserv. Gen. Res. 2, 231–234. 10.1007/s12686-010-9245-y

[B63] Pearse D. E. Arndt A. D. Valenzuela N. Miller B. A. Cantarelli V. H. Sites J. W. (2006). Estimating population structure under nonequilibrium conditions in a conservation context: continent-wide population genetics of the giant Amazon River turtle, *Podocnemis expansa* (Chelonia; Podocnemididae). Mol. Ecol. 15, 985–1006. 10.1111/j.1365-294X.2006.02869.x 16599962

[B64] Piry S. Luikart G.„ Cornuet J. (1999). BOTTLENECK: a computer program for detecting recent reduction in the effective size using allele frequency data. J. Hered. 90, 502–503. 10.1093/jhered/90.4.502

[B65] Pritchard J. K. Stephens M. Donnelly P. (2000). Inference of population structure using multilocus genotype data. Genetics 155, 945–959. 10835412 10.1093/genetics/155.2.945 PMC1461096

[B66] Putman A. I. Carbone I. (2014). Challenges in analysis and interpretation of microsatellite data for population genetic studies. Ecol. Evol. 4, 4399–4428. 10.1002/ece3.1305 25540699 PMC4267876

[B67] R Development Core Team (2011). R: A Language and Environment for Statistical Computing. Vienna: R Foundation for Statistical Computing Available online at: www.R-project.org

[B68] Reis R. E. Albert J. S. Di Dario F. Mincarone M. M. Petry P. Rocha L. A. (2016). Fish biodiversity and conservation in South America. J. Fish Biol. 89, 12–47. 10.1111/jfb.13016 27312713

[B69] Rice W. R. (1989). Analyzing tables of statistical tests. Evolution 43, 223–225. 28568501 10.1111/j.1558-5646.1989.tb04220.x

[B70] Rodrigues F. C. Farias I. P. Batista J. S. Alves-Gomes J. (2009). Isolation and characterization of microsatellites loci for “piramutaba” (*Brachyplatystoma vaillantii*, Siluriformes: Pimelodidae), one of the commercially most important migratory catfishes in the Amazon Basin. Conserv. Genet. Res. 1:365 10.1007/s12686-009-9084-x

[B71] Sambrook J. Fritsch E. F. Maniatis T. (1989). Molecular Cloning: A Laboratory Manual. Cold Springs Harbor, NY: Cold Springs Harbor Laboratory Press.

[B72] Santos M. C. F. Ruffino M. L. Farias I. P. (2007). High levels of genetic variability and panmixia of the tambaqui *Colossoma macropomum* (Cuvier, 1818) in the main channel of the Amazon River. J. Fish Biol. 71A, 33–44. 10.1111/j.1095-8649.2007.01514.x

[B73] Santos M. D. Hrbek T. Farias I. P. (2009). Microsatellite markers for the tambaqui (*Colossoma macropomum, Serrasalmidae, Characiformes*), an economically important keystone species of the Amazon River floodplain. Mol. Ecol. Resour. 9, 874–876. 10.1111/j.1755-0998.2008.02331.x 21564774

[B74] Schneider S. Excoffier L. (1999). Estimation of past demographic parameters from the distribution of pairwise differences when the mutation rates vary among sites: application to human mitochondrial DNA. Genetics 152, 1079–1089. Available online at: http://www.genetics.org/cgi/content/abstract/152/3/1079 10388826 10.1093/genetics/152.3.1079 PMC1460660

[B75] Sioli H. (1984). The Amazon and its main affluents: hydrography, morphology of the river courses and river types, in The Amazon, Limnology and Landscape Ecology of a Mighty Tropical River and its Basin, ed Sioli H. (New York, NY: Springer Verlag), 127–165.

[B76] Slatkin M. Hudson R. R. (1991). Pairwise comparisons of mitochondrial DNA sequences in stable and exponentially growing populations. Genetics 129, 555–562. 1743491 10.1093/genetics/129.2.555 PMC1204643

[B77] Storz J. F. Beaumont M. A. Alberts S. C. (2002). Genetic evidence for long-term population decline in a savannah-dwelling primate: inferences from a hierarchical bayesian model. Mol. Biol. Evol. 19, 1981–1990. 10.1093/oxfordjournals.molbev.a004022 12411607

[B78] Tajima F. (1989). Statistical method for testing the neutral mutation hypothesis by DNA polymorphism. Genetics 123, 585–595. 2513255 10.1093/genetics/123.3.585 PMC1203831

[B79] Tamura K. Stecher G. Peterson D. G. Filipski A. Kumar S. (2013). MEGA6: molecular evolutionary genetics analysis version 6.0. Mol. Biol. Evol. 30, 2725–2729. 10.1093/molbev/mst197 24132122 PMC3840312

[B80] Van Oosterhout C. Hutchinson W. F. Wills D. P. M. Shipley P. (2004). MICRO-CHECKER: software for identifying and correcting genotyping errors in microsatellite data. Mol. Ecol. Notes 4, 535–538. 10.1111/j.1471-8286.2004.00684.x

[B81] Venticinque E. Forsberg B. Barthen B. R. Petry P. Hess L. Mercado A. (2016). An explicit GIS-based river basin framework for aquatic ecosystem conservation in the Amazon. Earth Syst. Sci. Data 8, 651–661. 10.5194/essd-2016-17

[B82] Wang X. Lawrence Edwards R. Auler A. S. Cheng H. Kong K. Wang Y.. (2017). Hydroclimate changes across the Amazon lowlands over the past 45,000 years. Nature 541, 204–207. 10.1038/nature20787 28079075

[B83] Waples R. S. Do C. (2008). Ldne: a program for estimating effective population size from data on linkage disequilibrium. Mol. Ecol. Res. 8, 753–756. 10.1111/j.1755-0998.2007.02061.x 21585883

[B84] Wright S. (1965). The interpretation of population structure by F-statistics with special regard to systems of mating. Evolution 19, 395–420.

