# Supplementary material for: A Multilocus Approach to Understanding Historical and Contemporary Demography of the Keystone Floodplain Species Colossoma macropomum (Teleostei: Characiformes)
Source: Front Genet. 2018 Aug 14;9:263. doi: 10.3389/fgene.2018.00263 (PMC6102471; doi:10.3389/fgene.2018.00263)
Supplement: Supplementary file 1 [file Table_1.doc]

Supplementary Table S1 – Primers used for amplification and sequencing of the control region and ATPase gene of the mitochondrial DNA of *Colossoma macropomum*.

| *D-LOOP* | | |
| --- | --- | --- |
| *Primers* | *Primers sequences* | References |
| Chara_LDloop | 5’CCCACCACTAACTCCCAAAG 3’ | Present study |
| Chara_RDloop | 5’GGTTTTGGGGTTTGAGAGG 3’ | Present study |
| CMF2 | 5’CATCTGGTTCCTATTTCAGG 3’ | Present study |
| CMR2 | 5’GTGACCAAATGTCAGGTGGA 3’ | Present study |
| ATPase | | |
| *Primers* | *Primers sequences* | References |
| ATP 8.2_L8331 | 5’AAAGCRTYRGCCTTTTAAGC 3’ | Sivasundar *et al*. (2001) |
| CO3.2_H9236 | 5’GTTAGTGGTCAKGGGCTTGGRTC3’ | Sivasundar *et al*. (2001) |
