# Supplementary material for: A Multilocus Approach to Understanding Historical and Contemporary Demography of the Keystone Floodplain Species Colossoma macropomum (Teleostei: Characiformes)
Source: Front Genet. 2018 Aug 14;9:263. doi: 10.3389/fgene.2018.00263 (PMC6102471; doi:10.3389/fgene.2018.00263)
Supplement: Supplementary file 2 [file Table_2.doc]

Supplementary Table S2 – Multiplex system used in genotyping PCRs of *Colossoma* *macropomum* samples.

| Multiplex | Primers | Fluorescence | Size (pb) |
| --- | --- | --- | --- |
| Group 1 | Cm2B1 F-CAGGCAGTTAGAACGAGTTCAC  R-GCCTCCACACTGACACTTCA  Cm1E3 F-CTGACCGATGCAACACTCC  R-CCTGAACCAGGCTCCAGAT  Cm1H8 F-GGTCAAAACACACGCCACTA  R-CAGCAGACAGTAGGCAGAGAG | FAM  HEX  FAM | 120-170  200-250  250-320 |
| Group 2 | Cm2G5 F-GTAGCCGTCCCGTCCCTA  R-GCTCTCACAATATCTCTCACTGTC  Cm1D1 F-GCAAATGTGCACACCAATAG  R-GCAGAAGGTGAAGAGTCTTGTG  Cm1C6 F-TAAAGCCAAGCCAGGTCTGT  R-CGAAACATCAGGCACGAATA | FAM  HEX  FAM | 100-150  150-200  250-300 |
| Group 3 | Cm1A8 F-TGCTCTCCTGCAGTCTCTCA  R-TCATGGTTGCCACTCATCTC  Cm1G7 F-CCCAGCCTACTACAGGGTCA  R-CACACATCGCTCTTCTCTCC | FAM  FAM | 150-200  300-350 |
| Group 4 | Cm1F7 F-CCTATTGTGATGGCAGAGAGAG  R-CACAGACGTGTACAGCTGGTT  Cm1A11 F-CCAGCGGGTTAAAGCCTAC  R-GCAGCCTCACTGATACGTTG | HEX  FAM | 200-220  220-260 |
| Group 5 | Cm1F4 F-GGGGTGAGTCATTGAGGAGA  R-CCGATCATGAAGGCAGTCTC  Cm1F5 F-TTTCGGGAGCAGGACAAG  R-CAGTTAAGGGACCCAGTGGA | FAM  HEX | 200-250  250-300 |
| Group 6 | Cm1B8 F-CACAACCCACCTGTTTGATT  R-CTAATAACAAACCTACTTCCACTTCTC  Cm1C8 F-AGCATGTGTGGAACGTAGGG  R-CTGCCGATCACAGCACTAGA | FAM  HEX | 120-160  220-260 |
