# Supplementary material for: A Multilocus Approach to Understanding Historical and Contemporary Demography of the Keystone Floodplain Species Colossoma macropomum (Teleostei: Characiformes)
Source: Front Genet. 2018 Aug 14;9:263. doi: 10.3389/fgene.2018.00263 (PMC6102471; doi:10.3389/fgene.2018.00263)
Supplement: Supplementary file 6 [file Table_6.doc]

Supplementary Table S6 – Genetic parameters estimated for *C. macropomum* to test the isolation-with-migration model in IMa2 inferred from microsatellites data. θ =4Neµ (θ_1_ - locality 1, θ_2_ - locality 2, θ_A_ - ancestral of two localities); Generation time of separation between the two locations *t* = T / μ; Migration rate m = m / μ. Mutation rate μ = 10^-4^ / generation time. Generation time is assuming three years. Values in parentheses = 95% confidence limits.

| Localities | θ _1_ | θ _2_ | θ _A_ | *t* | *m*1→2 | *m*2→1 |
| --- | --- | --- | --- | --- | --- | --- |
| 1-Mexiana and 2- Tabatinga | 5.215  (0.73 - 63.17) | 5.845  (19.57 - 51.70) | 36.37  (0.52 - 56.73) | 0.0005  (0.0 - 0.39) | 4.27  (0.0 - 49.98) | 0.37  (1.50-49.98) |
| 1-Jacareacanga and 2- Main channel | 7.805  (2.62 - 17.11) | 3.955  (0.80 - 9.13) | 25.52  (16.62 - 39.66) | 0.1555  (0.01 - 0.81) | 0.08  (0.0 - 5.87) | 1.97  (0.0 - 15.97) |
| 1-Guajará-Mirim and 2- Main channel | 1.365  (0.31 - 2.97) | 6.055  (1.36 - 11.65) | 28.59  (19.36 - 44.98) | 0.0925  (0.005 - 0.62) | 4.92  (0.12 - 21.07) | 0.08  (0.0 - 4.67) |
| 1- Boca do Acre and 2- Main channel | 14.380  (5.91 - 65.69) | 4.095  (0.59 - 10.26) | 29.86  (20.27 - 49.17) | 0.0445  (0.002 - 0.86) | 4.42  (0.0 - 15.82) | 0.03  (0.0 - 22.77) |
| 1-Eirunepé and 2- Main channel | 4.795  (1.85-10.19) | 6.125  (2.55 - 11.45) | 28.52  (18.66 - 48.76) | 0.2235  (0.05 - 0.90) | 0.93  (0.0 - 6.57) | 0.07  (0.0 - 5.22) |
