# Supplementary material for: A Multilocus Approach to Understanding Historical and Contemporary Demography of the Keystone Floodplain Species Colossoma macropomum (Teleostei: Characiformes)
Source: Front Genet. 2018 Aug 14;9:263. doi: 10.3389/fgene.2018.00263 (PMC6102471; doi:10.3389/fgene.2018.00263)
Supplement: Supplementary file 7 [file Table_7.doc]

Supplementary Table S7 – Genetic parameters estimated for *C. macropomum* in MIGRATE version 3.1.3 inferred from microsatellites data. Migration rate *M*=m/µ. Mutation rate µ=10^-4^/generation time (three years). θ =4*Ne*µ.

| M_2(1-21)_>M_1(M1-M21)_ | | | | | | | | | | | | | | | | | | | | | | |
| --- | --- | --- | --- | --- | --- | --- | --- | --- | --- | --- | --- | --- | --- | --- | --- | --- | --- | --- | --- | --- | --- | --- |
| θ | M_1_/M_2_ | Mex | Alm | San | Ita | Jac | Ori | Nha | Pin | Bor | Hum | Pve | Gua | Mao | Tap | Bda | Coa | Tef | Car | Eir | Fbo | Tab |
| 18.55 | Mex | - | 1.57 | 1.62 | 1.72 | 2.07 | 1.12 | 1.77 | 2.47 | 1.27 | 1.47 | 0.67 | 1.87 | 0.32 | 1.32 | 1.77 | 2.07 | 1.32 | 0.72 | 3.12 | 1.32 | 1.47 |
| 5.35 | Alm | 1.67 | - | 1.27 | 0.82 | 2.12 | 1.42 | 2.32 | 3.27 | 0.92 | 1.87 | 1.02 | 0.87 | 2.22 | 0.72 | 1.77 | 2.27 | 0.62 | 1.77 | 1.12 | 0.82 | 2.02 |
| 3.95 | San | 1.22 | 1.07 | - | 1.57 | 1.37 | 1.37 | 1.37 | 0.92 | 1.52 | 1.52 | 0.27 | 0.52 | 1.22 | 0.87 | 1.97 | 3.12 | 0.87 | 0.97 | 2.52 | 2.17 | 1.82 |
| 1.45 | Ita | 1.72 | 0.42 | 1.42 | - | 1.22 | 3.02 | 0.55 | 2.27 | 1.97 | 1.47 | 2.42 | 1.02 | 1.07 | 0.47 | 2.72 | 3.12 | 4.17 | 2.57 | 1.77 | 1.82 | 1.97 |
| 0.45 | Jac | 1.77 | 1.22 | 2.17 | 1.12 | - | 2.17 | 4.47 | 1.72 | 0.27 | 1.42 | 2.07 | 0.47 | 1.62 | 2.97 | 0.67 | 1.87 | 4.27 | 1.82 | 1.27 | 0.72 | 1.22 |
| 3.95 | Ori | 1.12 | 1.47 | 2.02 | 1.02 | 0.77 | - | 1.52 | 1.22 | 3.12 | 1.92 | 2.67 | 0.87 | 1.57 | 1.12 | 1.72 | 1.57 | 0.87 | 1.37 | 0.52 | 1.47 | 2.17 |
| 4.45 | Nha | 2.52 | 1.82 | 1.72 | 0.77 | 1.92 | 2.22 | - | 2.62 | 2.52 | 1.02 | 1.07 | 1.27 | 1.02 | 1.17 | 1.12 | 1.32 | 1.77 | 0.97 | 0.27 | 1.22 | 1.07 |
| 3.25 | Pin | 1.27 | 1.07 | 2.52 | 1.02 | 0.62 | 1.87 | 1.97 | - | 3.62 | 2.32 | 2.82 | 1.87 | 1.17 | 1.42 | 2.02 | 1.42 | 1.37 | 2.62 | 0.77 | 0.87 | 1.17 |
| 2.35 | Bor | 1.52 | 0.67 | 0.97 | 1.17 | 2.82 | 0.62 | 2.37 | 2.02 | - | 3.02 | 2.02 | 0.92 | 1.92 | 0.62 | 2.02 | 1.37 | 1.22 | 4.07 | 0.57 | 1.07 | 0.92 |
| 0.65 | Hum | 1.67 | 3.12 | 1.22 | 2.92 | 1.62 | 1.57 | 3.52 | 1.12 | 3.37 | - | 0.37 | 0.77 | 2.47 | 0.67 | 1.47 | 3.42 | 1.32 | 0.72 | 1.12 | 1.82 | 1.92 |
| 0.55 | Pve | 1.87 | 2.07 | 0.02 | 2.92 | 1.22 | 1.12 | 0.92 | 1.42 | 1.77 | 2.02 | - | 1.02 | 1.22 | 1.42 | 0.87 | 2.67 | 2.42 | 1.67 | 0.62 | 1.87 | 1.22 |
| 0.85 | Gua | 1.82 | 1.67 | 2.02 | 2.32 | 1.92 | 1.67 | 7.17 | 1.27 | 1.42 | 2.57 | 1.92 | - | 1.62 | 0.77 | 1.07 | 3.02 | 1.07 | 3.17 | 2.67 | 1.77 | 1.02 |
| 1.65 | Mao | 0.92 | 2.82 | 1.47 | 4.77 | 1.52 | 1.72 | 0.42 | 2.57 | 5.12 | 1.42 | 1.07 | 1.07 | - | 1.27 | 0.77 | 0.37 | 2.22 | 2.47 | 1.42 | 1.17 | 1.47 |
| 7.05 | Tap | 0.67 | 1.07 | 0.37 | 0.37 | 0.82 | 1.17 | 1.37 | 1.77 | 0.52 | 1.12 | 2.27 | 0.67 | 1.77 | - | 3.42 | 1.52 | 0.72 | 2.47 | 1.92 | 1.07 | 1.77 |
| 1.25 | Bda | 1.42 | 1.07 | 1.32 | 0.47 | 0.97 | 0.92 | 1.22 | 1.27 | 1.37 | 0.67 | 1.92 | 0.52 | 2.77 | 1.07 | - | 1.32 | 0.87 | 0.57 | 0.62 | 0.57 | 0.97 |
| 2.65 | Coa | 1.02 | 1.27 | 2.57 | 1.02 | 1.22 | 2.97 | 0.32 | 2.77 | 0.87 | 1.12 | 1.97 | 1.42 | 2.37 | 1.97 | 0.92 | - | 1.92 | 0.97 | 3.62 | 1.62 | 1.42 |
| 2.75 | Tef | 1.57 | 0.32 | 1.07 | 0.47 | 1.02 | 0.87 | 3.97 | 0.67 | 1.02 | 1.77 | 0.97 | 1.17 | 1.12 | 0.62 | 0.02 | 1.27 | - | 2.12 | 1.17 | 2.37 | 1.82 |
| 4.45 | Car | 0.32 | 1.32 | 2.82 | 4.17 | 1.02 | 1.62 | 1.72 | 0.07 | 1.52 | 1.07 | 1.57 | 1.32 | 3.17 | 1.42 | 1.37 | 3.62 | 1.57 | - | 1.22 | 1.27 | 1.32 |
| 0.55 | Eir | 1.32 | 0.77 | 0.57 | 1.07 | 1.17 | 1.32 | 3.57 | 0.97 | 0.27 | 5.12 | 2.32 | 1.92 | 0.32 | 2.52 | 0.97 | 1.12 | 1.32 | 1.62 | - | 1.82 | 0.52 |
| 6.45 | Fbo | 2.97 | 1.87 | 2.32 | 2.82 | 1.77 | 1.52 | 1.27 | 0.77 | 1.17 | 0.57 | 1.97 | 0.42 | 1.92 | 2.02 | 1.72 | 2.17 | 0.77 | 1.82 | 1.07 | - | 1.42 |
| 3.05 | Tab | 3.62 | 3.52 | 2.37 | 0.97 | 0.62 | 4.12 | 2.62 | 2.82 | 2.92 | 1.37 | 2.32 | 1.07 | 3.62 | 0.97 | 2.12 | 2.87 | 2.32 | 0.32 | 1.27 | 1.12 | - |
|  | | | | | | | | | | | | | | | | | | | | | | |

Nota: Mex (Mexiana), Alm (Almeirim), San (Santarém), Ita (Itaituba), Jac (Jacareacanga), Ori (Oriximiná), Nha (Nhamundá), Pin (Parintins), Bor (Borba), Hum (Humaitá), Pve (Porto Velho), Gua (Guajará-Mirim), Mao (Manaus), Tap (Tapauá), Bda (Boca do Acre), Coa (Coari), Tef (Tefé), Car (Carauari), Eir (Eirunepé), Fbo (Fonte Boa) e Tab (Tabatinga).
